# Root and branch hydraulic functioning and trait coordination across organs in drought-deciduous and evergreen tree species of a subtropical highland forest

**DOI:** 10.3389/fpls.2023.1127292

**Published:** 2023-06-12

**Authors:** Marian Schönauer, Peter Hietz, Bernhard Schuldt, Boris Rewald

**Affiliations:** ^1^ Department of Forest and Soil Sciences, Institute of Forest Ecology, University of Natural Resources and Life Sciences, Vienna, Austria; ^2^ Department of Forest Work Science and Engineering, Department of Forest Sciences and Forest Ecology, Georg-August-Universität Göttingen, Göttingen, Germany; ^3^ Department of Integrative Biology and Biodiversity Research, Institute of Botany, University of Natural Resources and Life Sciences Vienna, Vienna, Austria; ^4^ Chair of Forest Botany, Institute of Forest Botany and Forest Zoology, Technical University of Dresden, Tharandt, Germany

**Keywords:** Ethiopian Highland, leaf habits, root and branch hydraulics, seasonally dry subtropical forest, wood density, xylem-specific conductivity

## Abstract

Vessel traits are key in understanding trees’ hydraulic efficiency, and related characteristics like growth performance and drought tolerance. While most plant hydraulic studies have focused on aboveground organs, our understanding of root hydraulic functioning and trait coordination across organs remains limited. Furthermore, studies from seasonally dry (sub-)tropical ecosystems and mountain forests are virtually lacking and uncertainties remain regarding potentially different hydraulic strategies of plants differing in leaf habit. Here, we compared wood anatomical traits and specific hydraulic conductivities between coarse roots and small branches of five drought-deciduous and eight evergreen angiosperm tree species in a seasonally dry subtropical Afromontane forest in Ethiopia. We hypothesized that largest vessels and highest hydraulic conductivities are found in roots, with greater vessel tapering between roots and equally-sized branches in evergreen angiosperms due to their drought-tolerating strategy. We further hypothesized that the hydraulic efficiencies of root and branches cannot be predicted from wood density, but that wood densities across organs are generally related. Root-to-branch ratios of conduit diameters varied between 0.8 and 2.8, indicating considerable differences in tapering from coarse roots to small branches. While deciduous trees showed larger branch xylem vessels compared to evergreen angiosperms, root-to-branch ratios were highly variable within both leaf habit types, and evergreen species did not show a more pronounced degree of tapering. Empirically determined hydraulic conductivity and corresponding root-to-branch ratios were similar between both leaf habit types. Wood density of angiosperm roots was negatively related to hydraulic efficiency and vessel dimensions; weaker relationships were found in branches. Wood density of small branches was neither related to stem nor coarse root wood densities. We conclude that in seasonally dry subtropical forests, similar-sized coarse roots hold larger xylem vessels than small branches, but the degree of tapering from roots to branches is highly variable. Our results indicate that leaf habit does not necessarily influence the relationship between coarse root and branch hydraulic traits. However, larger conduits in branches and a low carbon investment in less dense wood may be a prerequisite for high growth rates of drought-deciduous trees during their shortened growing season. The correlation of stem and root wood densities with root hydraulic traits but not branch wood points toward large trade-offs in branch xylem towards mechanical properties.

## 1 Introduction

As tree performance is typically linked to resource availability and utilization, related traits receive specific attention (Reich, 2014; Rosas et al., 2019; Khan et al., 2020). Ethiopia has been identified as a hotspot of increasing drought frequencies under climate change (Spinoni et al., 2019), rendering knowledge on acquisitive and economic traits of tree genetic resources key for understanding the impact of climate change on these forest ecosystems (Skelton et al., 2019). Evolutional lineage (e.g., gymnosperms, angiosperms), species-specific ecological strategies, and effective water availabilities underlie differences in trees’ hydraulic structures (Anderegg et al., 2016). For example, when the water availability is non-limiting, wider vessels with a higher hydraulic efficiency usually favor higher growth rates (Hoeber et al., 2014; Kotowska et al., 2015; Schumann et al., 2019). However, adaptations to drought have been found to modify this relationship (Méndez-Alonzo et al., 2012; Worbes et al., 2013; Rosas et al., 2019). For example, by shedding their leaves after the onset of the dry season, drought-deciduous species avoid developing a very negative water potential and thus may not require a very drought resistant xylem (Markesteijn et al., 2011; Souza et al., 2020). In contrast, evergreen trees in tropical climates with a pronounced dry season tend to have a high wood density, lower hydraulic conductivity, narrower vessels, and lower xylem vulnerability (Choat et al., 2005; Vinya et al., 2012; Kröber et al., 2014; Ribeiro et al., 2022). However, categorizing tree species solely based on their deciduousness, or leaf phenology, may be an oversimplification, as multiple leaf and wood traits influence hydraulic patterns (Souza et al., 2020; Ribeiro et al., 2022). In agreement, Hoeber et al. (2014) observed no differences in branch vessel diameter or hydraulic efficiency between leaf habits in young trees in the seasonally dry tropics. However, it remains largely unknown whether (mature) trees of species differing in leaf habit vary systematically in their root or branch vascular architecture in seasonally dry tropical ecosystems—as one might speculate based on their different drought resistance mechanisms, i.e. drought-avoidance (drought-deciduous) vs. drought-tolerance (evergreens).

Since tree performance depends on coordinated functioning, traits are generally considered to be related among organs (Weemstra et al., 2022), to ensure, for instance, that the evaporative demands of leaves is met by respective transport and acquisitive traits at upstream sections of the hydraulic pathway (Eissenstat and Yanai, 2002). Furthermore, friction and gravitation increasingly constrain water flow with progressing length of flow paths and height, respectively, causing lower xylem water potentials in leaves and upper stem sections compared to roots and basal stem sections (Fulton et al., 2014; Olson et al., 2021). To compensate this, xylem conduits of temperate tree species frequently taper along the hydraulic pathway from coarse roots to leaves (Mencuccini et al., 2007; Anfodillo et al., 2013; Lübbe et al., 2022), while vessel frequencies increase (Lintunen and Kalliokoski, 2010; Lübbe et al., 2022). Conduits might not taper continuously from roots to branches, but trees in tropical perhumid environments, for example, were shown to hold a hump-shaped vessel diameter distribution with largest vessels observed at the stem base (Schuldt et al., 2013; Kotowska et al., 2015). Similarly, the ‘Widened Pipe Model’ predicts that stem xylem conduits should be narrowest at most upper stem sections, widening quickly before plateauing towards the stem base (Koçillari et al., 2021; Olson et al., 2021). By this model, within-individual vessel widening, as well as across-individual conduit diameters at the stem base could be predicted (Olson et al., 2021), with diameter at stem base increasing with tree height. However, ratios of coarse root to branch sapwood areas (Kotowska et al., 2015), or organ-specific tissue differentiation processes (Ye, 2002) might further underlie hydraulic differences between organs. For example, Longui et al. (2017) reported conduit diameters in 5-10 years old trees of five Cerrado species to decrease strongly from roots to the stem base, then increasing towards the stem top and plateauing in branches, while conduit frequencies generally increased towards branches in young trees of five species in the Brazilian Cerrado (but see Machado et al. (2007) and Longui et al. (2012) for contrasting results in the same ecosystem). To our knowledge, the degree of vessel tapering has not yet been determined for species differing in leaf habit from seasonally dry (sub-)tropical environments. Because the branch xylem of evergreen angiosperms has been found to be composed of smaller vessels compared to drought-deciduous tree species (Choat et al., 2005), one might speculate that the degree of vessel diameter reduction from roots to branches is smaller in evergreen angiosperms (when tree height is comparable).

Furthermore, studies on species from moist tropical forests failed to detect relationships between vessel characteristics/hydraulic traits with wood density (WD) and suggested that any such relationship may be indirect (Poorter et al., 2010; Schuldt et al., 2013; Kotowska et al., 2015; Hietz et al., 2017). In contrast, a rather high correlation between stem hydraulics and WD was reported for tropical dry forests (Méndez-Alonzo et al., 2012; Hoeber et al., 2014). Few studies, e.g. in tropical moist (Fortunel et al., 2014), or temperate forests (Plavcová et al., 2019; Lübbe et al., 2022), have yet addressed the relation of stem WD with hydraulic properties and WD of distal (transport) organs below- and above-ground (i.e., coarse roots or branches).

An extensive number of studies have measured the variation in xylem hydraulic traits in trees along the flow path (Gleason et al., 2012; Schuldt et al., 2013; Kröber et al., 2014; Lübbe et al., 2022). However, seasonally dry (sub-)tropical ecosystems are underrepresented in global datasets, and information on xylem traits of Afromontane forest species remains particular rare while being key to develop a better understanding on species- and ecosystem functioning. While large, pristine Afromontane forests have largely disappeared, thousands of isolated stands remain around churches in the Ethiopian highlands (Wassie et al., 2005); the diversity of shrubs and trees in these ‘church forests’ is high (Aerts et al., 2016). In this work, we analyze similar-sized coarse roots and sun-exposed branches of 14 indigenous tree species of the Ethiopian Highland. We hypothesize that (i) vessel diameter is larger and xylem hydraulic efficiency is higher in similar-sized roots compared to branches in a subtropical forest exposed to seasonal drought, and that (ii) co-existing drought-deciduous and evergreen angiosperms differ in their hydraulic root-to-branch trait relations due to fundamentally different drought resistance mechanisms—with a lower relative vessel diameter reduction in branches of drought-deciduous compared to similar-sized evergreen trees. We further hypothesize (iii) that the hydraulic efficiencies of coarse roots and branches cannot be predicted from wood density—due to a varying dependency on conduit properties—but that wood densities of organs (coarse roots, stem and branches) are generally related within species.

## 2 Materials and methods

### 2.1 Study site

The study was conducted in the church forest Gelawdiwos (also Gelawdios)—located in the district Dera Woreda, Amhara National Regional State, north-central Ethiopia (11°38′25″N, 37°48′55″E). The forest covers c. 68 ha at c. 2500 meters above sea level. The forest is considered an old-growth, seasonally dry-evergreen Afromontane forest growing on slopes of 10-40° (Assefa et al., 2018; Belay et al., 2018), and is embedded in a mosaic of (degraded) grass- and croplands (Assefa et al., 2022). The climate of the mountainous study area is seasonally dry subtropical, with warm summers and dry winters (Köppen–Geiger classification ‘Cwb’, Peel et al., 2007). Annual precipitations ranges between 1200 and 1600 mm a^-1^, heavy rainfall occurring mainly from June to September (Summer) and occasional showers from March to May (Spring); the annual mean air temperature is c. 19°C (Abebe, 2017; Belay et al., 2018). Prevailing soil types are Cambisols; soil texture is 8-9% sand, 39-40% silt and 52% clay, pH(H_2_O) of 6.3, and c. 120 mg C g^-1^ and c. 11 mg N g^-1^ in the top soil (Eshete, 2007; Assefa et al., 2017).

### 2.2 Species selection

In the Gelawdiwos forest, at least 41 woody species of 29 families have been recorded, dominantly woody angiosperms (Wassie et al., 2010; Assefa et al., 2022). The only woody gymnosperm is *Afrocarpus falcatus* (syn. *Podocarpus falcatus*); at present occurring at relatively low density, it was likely more dominant in the past (Assefa et al., 2022). In 2014, the mean timber volume was estimated as 92.4 m^3^ ha^-1^ with an annual increment of 3.5 m^3^ ha^-1^ (Sisay et al., 2017). The density of trees with a diameter at breast height >10 cm was 315 ± 24 ha^-1^ and average tree height was 10.2 ± 0.7 m.

Fourteen frequent, native woody species (**Table 1**) of the Gelawdiwos forest were selected according to (assumed) abundance/relevance, following advice by local expert (Dessie Assefa, personal comment) and literature information (Eshete, 2007). Five species were deciduous and eight evergreen angiosperm trees, and one, in light of its assumed high natural abundance, the gymnosperm *A. falcatus*. Leaf habit classification of angiosperms followed literature information (**Table 1**); some species classified here as evergreen have, however, occasionally been described as semi-deciduous (e.g. *E. capensis* (Tilney et al., 2018) and *Prunus africana* (Seyoum et al., 2012)), and *C. macrostachyus* has been described as facultative deciduous (Seyoum et al., 2012). Within the study area, six plots similar in slope, canopy closure and tree height were established in August 2015. At each plot, one individual per species was selected based on following criteria: i) trees were in upper canopy layer, ii) lacked visible injuries, and iii) had no signs of recent disturbance in the surrounding. The minimum distance between plots was 20 m, in most cases c. 50-100 m; the individuals were thus treated as true replicates. Height of the selected trees was measured with a clinometer (Silva Sweden AB, Stockholm, Sweden); a tape was used for assessing diameter at breast height (**Table 1**).

**Table 1:** Studied woody species at the Gelawdiwos forest, Ethiopian highland, and the leaf habit, height (h), and diameter at breast height (DBH) of sampled individuals (mean ± SD, n=6).

Leaf habit	Species	Family	h (m)	DBH (cm)
Angiosperm, deciduous	*Albizia schimperiana* Oliv.^1^	Fabaceae	10 ± 2.5	25 ± 9
	*Bridelia micrantha* (Hochst.) Baill.^1,6^	Phyllanthaceae	8 ± 0.9	18 ± 4
	*Combretum molle* R.Br. ex G.Don^1^	Combretaceae	6 ± 1.1	10 ± 2
	*Croton macrostachyus* Hochst. ex Delile^1,5^	Euphorbiaceae	11 ± 4.5	20 ± 7
	*Schefflera abyssinica* (A. Rich.) Harms^3^	Araliaceae	16 ± 3.6	99 ± 35
	Mean		10 ± 4.5	34 ± 37
Angiosperm, evergreen	*Apodytes dimidiata* E. Mey. ex Arn.^1^	Icacinaceae	16 ± 3.1	105 ± 22
	*Calpurnia aurea* (Aiton) Benth.^1^	Fabaceae	8 ± 1.7	11 ± 3
	*Chionanthus mildbraedii* (Gilg & G. Schellenb.) Stearn^2^	Oleaceae	12 ± 6.5	59 ± 14
	*Dovyalis abyssinica* (A.Rich.) Warb.^3^	Salicaceae	7 ± 0.7	24 ± 12
	*Ekebergia capensis* Sparrm.^1^	Meliaceae	12 ± 2.5	52 ± 18
	*Maesa lanceolata* Forssk.^1^	Primulaceae	6 ± 1.8	12 ± 2
	*Prunus africana* (Hook.f.) Kalkman^1,5^	Rosaceae	10 ± 4.1	39 ± 27
	*Teclea nobilis* Delile^1^	Rutaceae	7 ± 0.9	21 ± 12
	Mean		10 ± 4.5	42 ± 34
Gymnosperm	*Afrocarpus falcatus* (Thunb.) C.N.Page^4.5^	Podocarpaceae	13 ± 2.9	21 ± 6

^1^Flora of Zimbabwe (Hyde et al., 2022), ^2^Central African Plants (Dressler et al., 2011), ^3^Flora of Zambia (Bingham et al., 2021), ^4^Flora of Mozambique (Hyde et al., 2022), ^5^ Seyoum et al. (2012), ^6^ Chidumayo (2005).

### 2.3 Sampling and storage

From each of the 84 tree individuals (n = 6 per species), two coarse root samples and three branches were collected. Root systems where carefully exposed, starting at the tree bole for correct identifications (Rewald et al., 2012), until a coarse root section with a diameter of c. 3-5 mm was discovered—usually at a soil depth of 10-30 cm. The positions of coarse root segments along the flow path (i.e., distance to tips, root order etc.; Freschet et al., 2021) remained unknown as large excavations were not tenable. Sun exposed branches (from the upper canopy) were collected by local tree climbers. For standardization, two-year old branch sections were identified and selected for all species; here, current-year sections were defined as zero-years-old. Subsequently, all distal leaves were collected and stored in plastic bags to prevent dehydration. Excising larger samples first, branch and root samples were recut to a length of 0.35 m, immediately rinsed in tap water and immersed in a silver chloride solution (Micropur, Katadyn, Germany) to reduce bacterial growth (Lübbe et al., 2022). The long branch was kept moist while being transported to the laboratory. Stem cores were collected from 13 tree species (n=2-6 per species) with a hand increment corer (5 mm diameter; Suunto, Vantaa, Finland) to determine wood density. Cores were extracted from the northern sector of the stem at breast height until reaching the center. Because of its very hard wood, no samples could be taken from *Teclea nobilis.* See **Supplementary Table S1** for a list of xylem traits, with acronyms and units, covered by this study.

### 2.4 Hydraulic conductivity

At the Amhara Regional Agricultural Research Institute (ARARI; Bahir Dar, Ethiopia) root and branch samples were submerged in tap water within 24 h after collection. Central and regularly shaped branch segments were shortened to a length of 50 mm and re-cut with a razor blade, while roots were cut to a length of 100 mm and likewise re-cut with a razor blade. The mean sample diameters were 2.8-4.9 mm in roots and 3.9-5.9 mm in branches (without bark; **Supplementary Table S2**).

Eighteen branch and 12 root samples were measured per species using the XYL’EM device (Bronkhorst, Montigny-Les-Cormeilles, France). Hydraulic conductance was measured following Sperry et al. (1988). Axial hydraulic conductivity (K_h_, kg m MPa^-1^ s^-1^) is given as mass flow rate (m s^-1^) per hydrostatic pressure gradient, created through a defined static head in the tubing system, and sample length (Δp l^-1^). The basal end of small branches (after debarking) and the distal end of coarse roots were attached to the tubing system. The mass flow rate was determined with a high precision flowmeter (Liquiflow, Instrutec, France; 0.2-10 g h^-1^) using degassed and filtered (0.2 µm) KCl solution (10 mM). For measuring the initial K_h_ (i.e., before flushing, to detect potential plugging of conduits) a hydrostatic pressure of 2 kPa was used for branches, and 1 kPa for roots. Xylem-specific conductivity ( ${\;}_{\text{KS}}^{\text{hydr}}$ , kg m^-1^ MPa^-1^ s^-1^) was calculated by dividing K_h_ by the xylem area (A_xylem_, m^2^). A_xylem_ was calculated as the area of an ellipse from two crosswise diameter measurements with a caliper ( ± 0.003 mm) at the basal (branch) or distal (root) end—minus the pith area in branches (**Supplementary Table S2**). The maximum xylem-specific conductivity ( ${\;}_{\text{KS}}^{\text{hydr}}$ ) was determined after flushing; branch samples were repeatedly flushed at 150 kPa for 30 s, root segments with a pressure of 200 kPa for 60 s until a stable value (k_max_) was reached (Martin-StPaul et al., 2014). Length, flushing pressures and time intervals were determined empirically during a pilot phase (data not shown). For *A. schimperiana* roots, no trustworthy ${\;}_{\text{KS}}^{\text{hydr}}$ values (i.e. ~3x greater values than in any other species, and a very high variability) could be obtained, probably due to leakages - these measurements were discarded.

Leaf area-specific conductivity (K_L_, kg m^-1^ MPa^-1^ s^-1^) of branches was calculated as maximum K_h_ (after flushing) per distal leaf area (m^2^, one-sided) per branch. See **Supplementary Methods** for details on leaf area, and K_L_.

### 2.5 Xylem anatomy, theoretical hydraulic conductivity and wood density

The coarse root and branch samples used above were labeled and conserved in 60% EtOH. For anatomical analyses, three samples per organ and species were selected, representing mean values of ${\;}_{\text{KS}}^{\text{hydr}}$ best. Subsequently, semi-thin cross sections (20 µm; at basal sides of branches and distal sides of roots) were cut using a sliding microtome (Reichert-Jung, Austria) and were embedded in Euparal (Carl Roth, Germany). Cross-sections were digitalized using a light microscope (Leica DM 5500B; Leica, Switzerland) equipped with a digital camera (Leica DMC 2900), and analyzed with the software ImageJ v. 1.37 (NIH, USA). Conduit lumen was estimated in a randomly selected radial sector (‘wedge’, opening angle of 20°-70°, bordered by ray parenchyma; (Hacke and Sperry, 2001)), with 57 ± 27 conduits per sector. The conduit density (CD, n mm^-2^) was calculated by relating n to the total area of the sector. The lumen fraction (F), i.e. the lumen-to-wood area ratio, was determined by relating conduit lumen area to total woody area of the respective radial sector. Mean conduit diameter (data not shown), hydraulically weighted conduit diameter (D_h_, µm), and potential conductivity per xylem area ( ${\;}_{\text{KS}}^{\text{pot}}$ , kg m^-1^ MPa^-1^ s^-1^) were calculated according to Sterck et al. (2008). ${\;}_{\text{KS}}^{\text{pot}}$ calculations used the Hagen–Poiseuille equation as $K_{S}^{pot}$ = ((π × ρ × ∑D^4^)/(128 η × A_xylem_)), where η is the viscosity (1.002 10^-9^ MPa s) and ρ the density of water (998.2 kg m^-3^) at 20°C. The Huber value (HV), i.e. the wood-to-leaf-area ratio, was calculated for branches by dividing the downstream leaf area (one-sided) by the area of sapwood (Tyree and Ewers, 1991).

Wood density (WD) was determined as the dry mass per fresh volume (g cm^-3^) of branch and coarse root segments used for measuring K_h_ (n = 12-18), as well as of the stem cores (n = 3-6). The bark was removed prior to determining wood density. Crosswise diameters at both sample ends and sample length were measured to ± 0.003 mm. Xylem volume (cm³) was calculated as the average of the elliptic woody area of both ends multiplied with the sample length, and corrected for the volume of the pith (in branches; **Supplementary Table S2**). The volume of stem cores was estimated using the corer inner dimension (5 mm) multiplied with the length of the core, measured after remoistening the cores for 12 h with tap water. WD_stem_ of *Teclea nobilis* could not be determined. The samples were dried at 100°C to a constant weight, and dry mass was determined to 0.1 mg.

### 2.6 Statistical analysis

Calculations were conducted with the free programming language R, version 4.2.0 (R Core Team, 2019), interfaced with Rstudio (version 2022.2.2.485, RStudio Team, 2016). Normal distribution of each trait was assessed via histograms and qqnorm plots; ${\;}_{\text{KS}}^{\text{hydr}}$ , ${\;}_{\text{KS}}^{\text{pot}}$ , and K_L_ were log-transformed for parametric tests. Data from different samples of the same tree were averaged per organ to avoid pseudo-replication. Linear models, followed by Post hoc Tukey tests were performed using the R package ‘emmeans’ (Lenth, 2022) to test for differences between species, organs and leaf habits. Letters indicating significant differences were created using the R package ‘multcomp’ (Hothorn et al., 2008). Pearson correlations of each trait between organs, as well as correlations of different traits within organs were calculated using species-wise means, and illustrated using the R package ‘corrmorant’ (Link, 2020). Root-to-branch ratios (R:B) of specific traits were calculated using either tree-wise means ( ${\;}_{\text{KS}}^{\text{hydr}}$ and WD) or species-wise means (for traits with fewer replicates). To investigate the impact of tree height on hydraulic traits, as studied by Olson et al. (2021) linear mixed-effects models (Bates et al., 2015) were fitted. These models were used to examine the correlation between D_h_ and ${\;}_{\text{KS}}^{\text{hydr}}$ with tree height, with species considered as a random effect. To calculate P-values for the linear mixed-effects models, we followed the method described in Kuznetsova et al. (2017). The significance level used for all tests was p<0.05 *, partially tendencies (p<0.1; (*)) are reported. Values are given as mean ± standard error (SE) or ± standard deviation (SD) as indicated.

## 3 Results

### 3.1 Xylem anatomy of roots and branches

**Figure 1:**
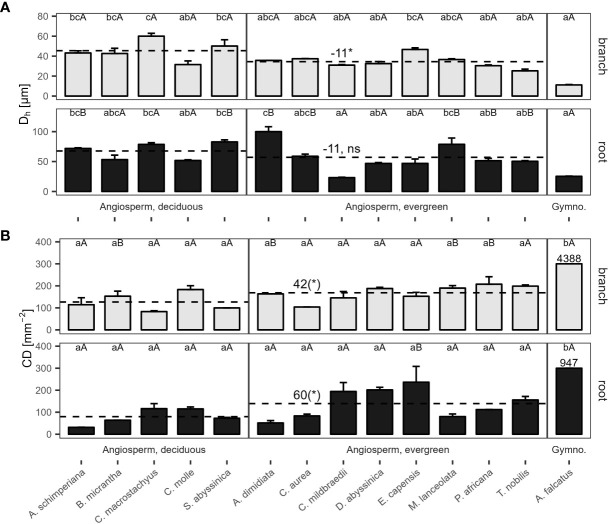
**(A)** Hydraulically weighted conduit diameter (D_h_), and **(B)** conduit density (CD) of 2^nd^-year branches (grey bars) and coarse roots (filled bars) of 14 woody species in a seasonally dry Ethiopian Highland forest. Species are deciduous or evergreen angiosperms, and the gymnosperm *A. falcatus*; see **Table 1** for details. Within traits and organs, small letters indicate significant differences between species, capital letters indicate differences between organs of the same species (Tukey test, mean+SE; n=3); dashed lines indicate means of deciduous and evergreen angiosperms, significant differences are denoted with a * (p<0.05), trends with a (*) (p<0.10); contrasts, calculated as marginal means of evergreen angiosperms minus marginal means of deciduous angiosperms (**Table 2**). Different y-axis scales of root and branch D_h_, and for CD of *A. falcatus*; ns, not significant.

**Figure 2:**
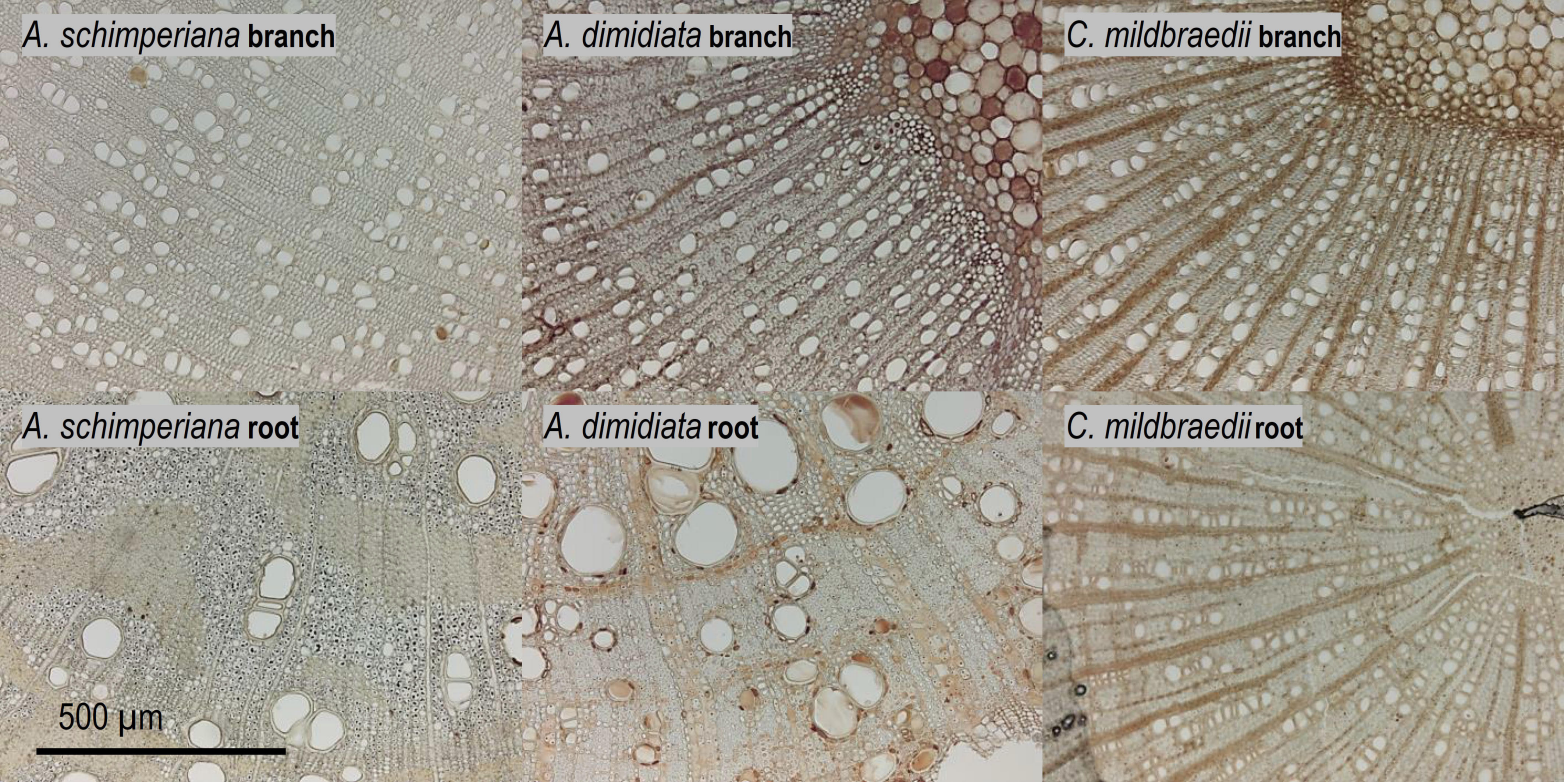
Xylem anatomy of 2^nd^-year branches (top) and coarse roots (bottom row) of the tree species *A. schimperiana, A. dimidiata* and *C. mildbraedii* in an Ethiopian Highland forest (**Table 1**). See **Supplementary Figures S1**, **S2** for cross sections of all species.

Hydraulically-weighted conduit diameters (D_h_) varied >4-fold across coarse roots (p<0.001, **Figure 1A**). D_h:root_ did not differ significantly between deciduous and evergreen angiosperms—holding mean D_h:root_ of 68 µm and 58 µm, respectively (**Table 2**). Yet, conduit density (CD_root_) of coarse roots tended to be lower in deciduous trees (p=0.099), where 80 conduits mm^-2^ were observed, compared to 139 n mm^-2^ in evergreen angiosperms (**Figure 1B**); the diameter of the measured roots was similar in both groups (3.82 ± 0.62 mm vs. 4.10 ± 0.44 mm in deciduous and evergreen species, respectively (mean ± SD); **Supplementary Table S2**). Angiosperms’ CD_root_ varied between 31 mm^-2^ in *A. schimperiana* and 236 mm^-2^ in *E. capensis*; CD_root_ of *A. falcatus* was 947 mm^-2^. The conduit lumen fraction (F_root_) ranged from 6.2% in *C. mildbraedii* to 36.8% in *C. macrostachyus* (**Supplementary Table S3**); mean F_root_ did not differ between leaf habits (21-22%, **Table 2**). In contrast to roots, the variation in D_h_ in branches was lower, with species-means between 25.3 µm (*T. nobilis*) and 60.0 µm (*C. macrostachyus*) (**Figure 1A**). D_h:branch_ of deciduous (45.5 µm) were significantly greater than in evergreen angiosperms (34.5 µm; **Table 2**; **Figure 1**); D_h:branch_ of *A. falcatus* was 11.1 µm. CD_branch_ varied widely among species, e.g. 83 mm^-2^ in *C. macrostachyus* to 208 mm^-2^ in *P. africana* (**Figure 1B**). F_branch_ of angiosperm species varied between 7.4% in *C. mildbraedii* and 18.2% in *E. capensis* (**Supplementary Table S3**)—evergreen and deciduous trees held similar F values (**Table 2**). F_branch_ in *A. falcatus* was 26.2%. See **Figure 2**; **Supplementary Figures S1**, **S2** for example cross sections.

**Table 2:** Contrasts among hydraulic traits of 13 deciduous and evergreen woody angiosperms in a seasonally dry Ethiopian Highland forest (deciduous – evergreen), calculated as marginal means of linear models.

Trait^§^	contrast	SE	df	t.ratio	p.value
D_h:root_	11	12	11	0.91	0.38
CD_root_	-60	33	11	-1.8	0.099
F_root_	-0.92	5.1	11	-0.18	0.86
$K_{S:root}^{hydr}$	1.5	0.54	10	0.63	0.54
$K_{S:root}^{pot}$	9.4	0.99	11	0.6	0.56
D_h:branch_	11	4.6	11	2.4	0.036
CD_branch_	-42	21	11	-2	0.071
F_branch_	2.1	2.1	11	1	0.32
CL	-0.054	0.15	11	-0.36	0.73
$K_{S:branch}^{hydr}$	1.1	0.37	11	1.1	0.30
$K_{S:branch}^{pot}$	8.4	0.85	11	2.3	0.045
K_L_	3.6	2.4	11	1.9	0.085
HV	0.0012	0.0016	11	0.79	0.45
WD_root_	-0.087	0.057	11	-1.5	0.16
WD_branch_	-0.024	0.051	11	-0.47	0.65
WD_stem_	-0.14	0.056	10	-2.5	0.031

^§^Subscripts ‘root’, ‘branch’ or ‘stem’ mark trait values of specific organs. Traits are: Hydraulically weighted conduit diameter (D_h_, µm), conduit density (CD, n mm^-2^), conduit lumen fraction (F, %), potential ( $K_{S}^{hydr}$ ), and hydraulic xylem-specific conductivity ( $K_{S}^{hydr}$ , kg m^-1^ MPa^-1^ s^-1^), conduit length (CL, m), leaf area-specific conductivity (K_L_, kg m^−1^ MPa^−1^ s^−1^) and wood density (WD, g cm^-3^) of coarse roots, branches and/or stem.

### 3.2 Specific hydraulic conductivity of roots and branches

**Figure 3:**
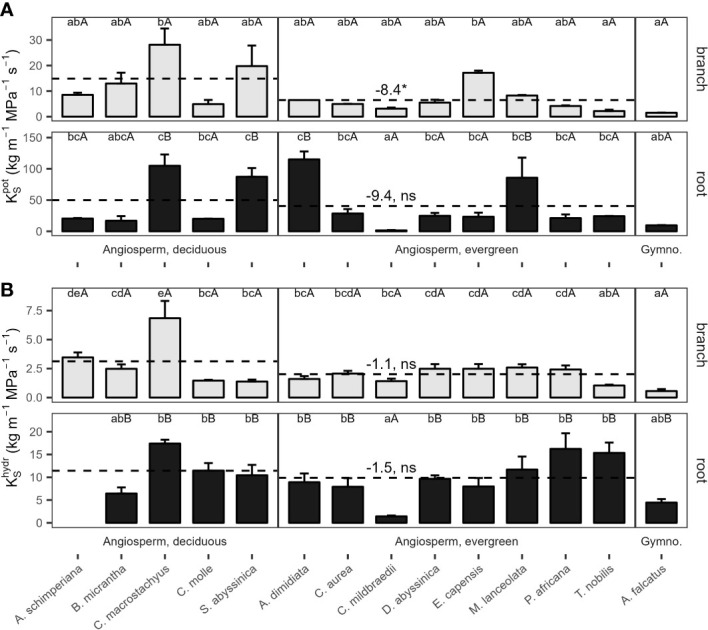
**(A)** Potential ( $K_{S}^{pot}$ ) and **(B)** measured ( $K_{S}^{hydr}$ ) specific conductivity of 2^nd^-year branches (grey bars) and coarse roots (filled bars) of 14 tree species in a seasonally dry Ethiopian Highland forest. Species are 13 deciduous or evergreen angiosperm trees and the gymnosperm *A. falcatus*; see **Table 1** for details. Small letters indicate significant differences between species among organs, capital letters indicate differences between organs of the same species; dashed lines indicate means of deciduous and evergreen Angiosperms, significant differences between means are denoted with a * (p<0.05), trends with a (*) (p<0.1); ns, not significant (Tukey test, Mean+SE; n( $K_{S}^{pot}$ )=3, n( $K_{S}^{hydr}$ )=6). Contrasts, calculated as marginal means of evergreen angiosperms minus marginal means of deciduous angiosperms, are given. Different y-axis scales of root and branch K_S_; see **Supplementary Figure S3** for ( $K_{S}^{pot}$ ) to ( $K_{S}^{hydr}$ ) relations; ( $K_{S}^{hydr}$ ) of *A. schimperiana* roots is not available.

Potential xylem-specific conductivities of coarse roots ( $K_{S:root}^{pot}$ ) ranged from 1.5 kg m^−1^ MPa^−1^ s^-1^ (*C. mildbraedii*), over 9.6 kg m^−1^ Mpa^−1^ s^-1^ (*A. falcatus*), to 115 kg m^−1^ MPa^−1^ s^-1^ (*A. dimidiata*; **Figure 3A**). *A. dimidiata* branches had a $K_{S:branch}^{pot}$ of 6.5 kg m^−1^ MPa^−1^ s^-1^, the average value among angiosperms was 9.1 kg m^−1^ MPa^−1^ s^-1^. Lowest $K_{S:branch}^{pot}$ were estimated for *A. falcatus* (1.5) and the evergreen *T. nobilis* (2.2 kg m^-1^ MPa^-1^ s^-1^). $K_{S:branch}^{pot}$ was significantly greater in deciduous as compared evergreen angiosperms (p=0.045, **Table 2**); however, some species showed a high deviation from leaf habit averages (**Figure 3A**). Empirically determined conductivity $K_{S:root}^{hydr}$ of angiosperms ranged between 1.4 kg m^-1^ MPa^-1^ s^-1^ in *C. mildbraedii*, to 17.4 kg m^-1^ MPa^-1^ s^-1^ in *C. macrostachyus* (**Figure 3B**). In general, $K_{S}^{hydr}$ was significantly lower in branches (2.3 kg m^−1^ MPa^−1^ s^-1^) compared to coarse roots (9.5 kg m^−1^ MPa^−1^ s^-1^; p<0.001, **Table 3**). Noteworthy, coarse roots and branches of e.g. *C. mildbraedii* had similar $K_{S}^{hydr}$ . $K_{S}^{hydr}$ was strongly positively correlated with $K_{S}^{pot}$ (p<0.001, R²=0.67, **Supplementary Figure S3**); $K_{S}^{hydr}$ was on average c. 25% of $K_{S}^{pot}$ .

**Table 3:** Linear models comparing hydraulic traits between coarse roots and branches of 13 woody angiosperms in a seasonally dry Ethiopian Highland forest.

Trait	contrast	SE	df	t.ratio	p.value
D_h_	22	6.2	24	3.6	0.0014
CD	-36	21	24	-1.7	0.096
F	9.3	2.6	24	3.6	0.0015
$K_{S}^{pot}$	34	11	24	3.1	0.0044
$K_{S}^{hydr}$	7.2	1.3	23	6.1	3.5e-06
WD	-0.11	0.038	24	-2.9	0.0079

Contrasts are calculated as marginal means of roots minus marginal means of branches per trait. Traits are: Hydraulically weighted conduit diameter (D_h_, µm), conduit density (CD, n mm^-2^), conduit lumen fraction (F, %), potential ( $K_{S}^{pot}$ ) and xylem-specific conductivity ( $K_{S}^{hydr}$ , kg m^-1^ MPa^-1^ s^-1^), and wood density (WD, g cm^-3^).

Leaf area-specific conductivities (K_L_) of angiosperms ranged from 313 kg m^−1^ MPa^−1^ s^−1^ in *C. aurea* to 16,368 kg m^−1^ MPa^−1^ s^−1^ in *A. schimperiana* (**Supplementary Table S3**). K_L_ tended to be greater in deciduous than in evergreen angiosperms (p=0.085; **Table 2**). Lowest Huber values (HV) were observed in *A. falcatus* (0.696 10^-4^ m^2^ m^-2^), followed by much greater values in evergreen (13.9 10^-4^ m^2^ m^-2^) and deciduous angiosperms (26.4 10^-4^ m^2^ m^-2^, **Supplementary Table S3**).

### 3.3 Hydraulic trait relations between and within organs

**Figure 4:**
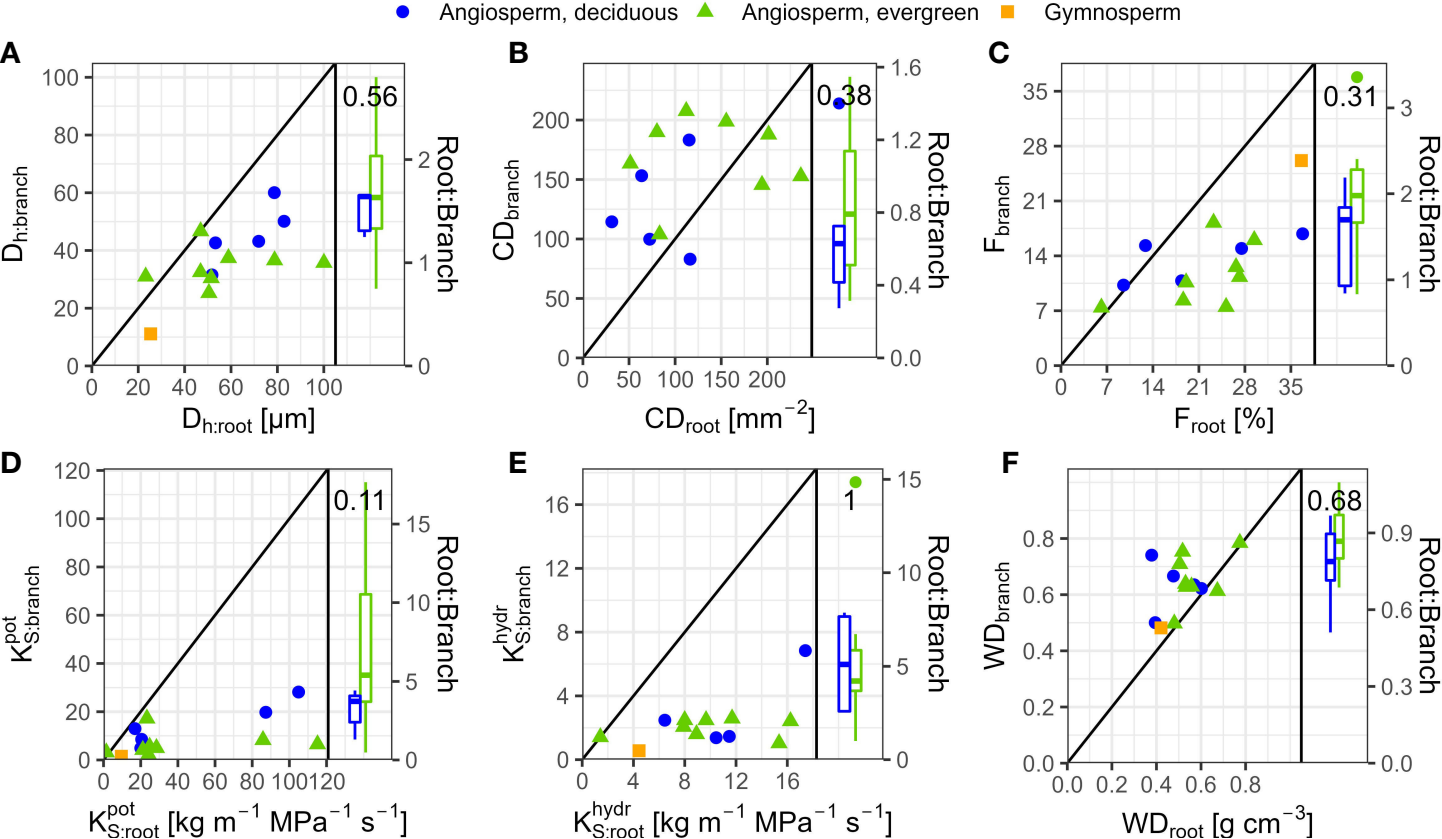
Comparison of anatomical and hydraulic traits between 2^nd^-year branches and coarse roots of 14 tree species in a seasonally dry Ethiopian Highland forest. Species are deciduous (blue) and evergreen (green) angiosperm trees and the gymnosperm *A. falcatus* (yellow; means per species); see **Table 1** for details. **(A)** D_h_, Hydraulically-weighted conduit diameter, **(B)** CD, Conduit density; *A. falcatus* has been omitted to increase readability, **(C)** F, Conduit lumen fraction, **(D)** $K_{S}^{pot}$ , Potential xylem-specific conductivity, **(E)** $K_{S}^{hydr}$ , Xylem-specific conductivity, and **(F)** WD, Wood density. Root-to-branch ratios (R:B) of traits are given as boxplots per leaf habit; see **Supplementary Table S4** for R:B means. Diagonal lines indicate R:B ratios of 1.

**Figure 5:**
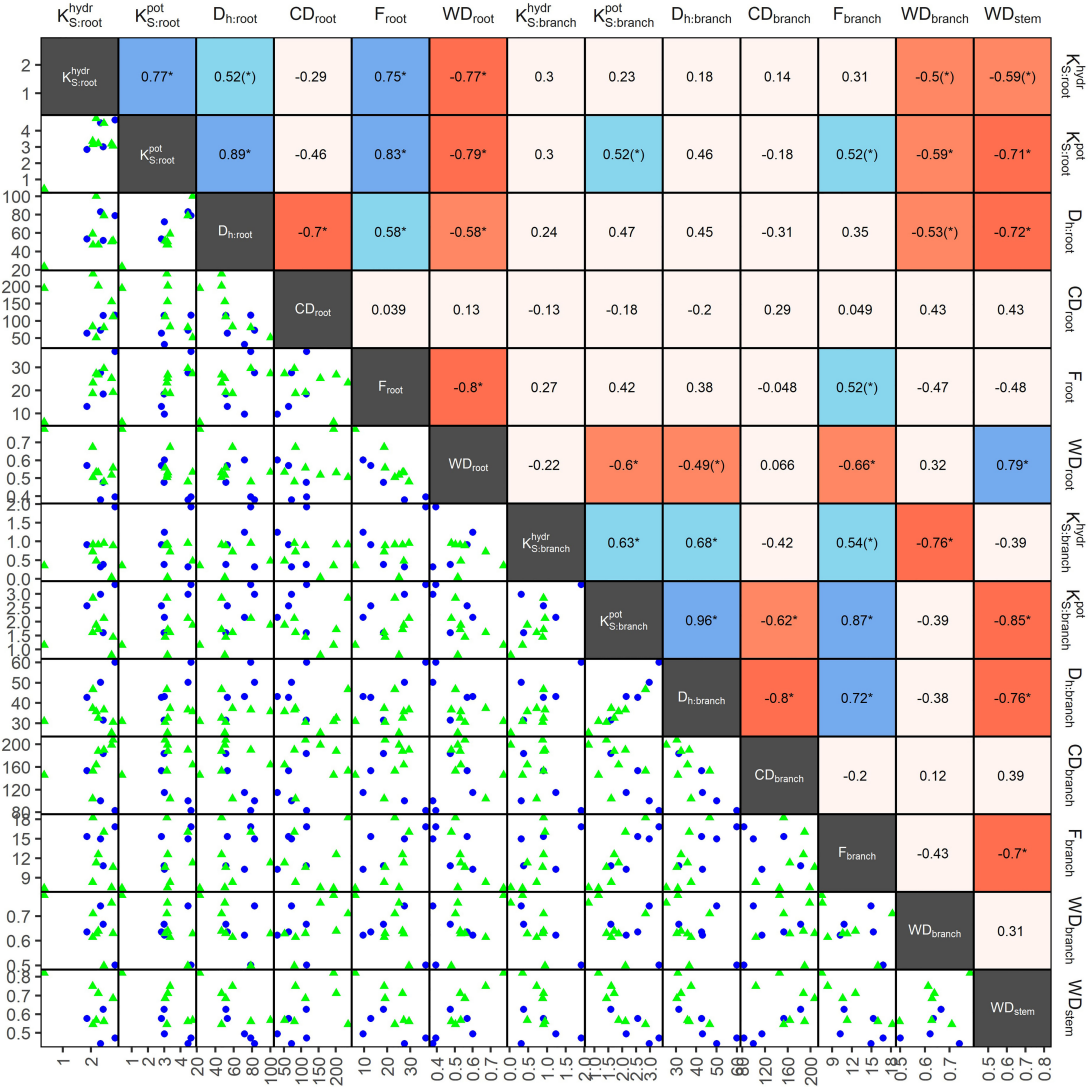
Pearson coefficients of correlation (upper triangles), and scatterplots (lower triangle) of anatomical and hydraulic traits of coarse roots and 2^nd^-year branches, and wood density (stem), of 13 deciduous (blue dots) and evergreen (green triangles) angiosperm tree species in a seasonally dry Ethiopian Highland forest; see **Table 1** for details. Significant correlations are colored, and levels of significance are indicated (* for p<0.05, (*) for p<0.1). Traits: hydraulically weighted conduit diameter (D_h_, µm), conduit density (CD, n mm^-2^), conduit lumen fraction (F, %), and potential ( $K_{S}^{pot}$ ) and measured specific conductivity ( $K_{S}^{hydr}$ ; kg m^-1^ MPa^-1^ s^-1^) of coarse root or branch samples; wood density of roots, branches and stems (WD, g cm^-3^). $K_{S}^{hydr}$ , $K_{S}^{pot}$ were log10-transformed, other traits were min-max-transformed.

In angiosperms, average D_h_, F, $K_{S}^{pot}$ , and $K_{S}^{hydr}$ in coarse roots were significantly greater than in branches; CD tended (p=0.096) to be lower in roots (**Table 3**). For example, D_h_ of angiosperm branches (39 µm) were on average 37% lower than those in roots (61 µm; p<0.001). These differences were similar between deciduous (D_h:root_ = 68 µm, D_h:branch_ = 46 µm) and evergreen (D_h:root_ = 57 µm, D_h:branch_ = 35 µm) angiosperms. In accordance, F_root_ was on average 9.3% greater than F_branch_ (p=0.002). Average root-to-branch ratios (R:B) of $K_{S}^{hydr}$ were comparable, with 5.1 for deciduous and 5.8 for evergreen angiosperms (**Figure 4**; **Supplementary Table S4**). Including *A. falcatus*, linear models applied on species-means revealed significant positive correlations between root and branch traits: D_h_ (p=0.026), CD (p<0.001), F (p=0.013), $K_{S}^{pot}$ (p=0.04), and a trend for $K_{S}^{hydr}$ (p=0.07; **Supplementary Table S5**). When calculated for angiosperms only, however, only marginally positive correlations were found (**Figure 4**; **Supplementary Table S5**). Pearson coefficients indicated clear correlations between some anatomical and hydraulic traits of angiosperms (**Figure 5**). In particular, $K_{S:root}^{hydr}$ showed a significant positive correlation to F_root_. $K_{S:branch}^{hydr}$ was significantly correlated to D_h:branch_. Correlations between the hydraulic traits of different organs were scarce and only trends were detected (**Figure 5**). Significant correlations were not observed between the hydraulic properties $K_{\left[S\right]}^{\left[hydr\right]}$ and D[_h_] of branches and roots and tree height (**Supplementary Table S6**).

Differences in wood densities (WD) were found (**Table 4**). For example, *C. mildbraedii* held the densest coarse roots (0.77 g cm^-3^) while *S. abyssinica* had a WD_root_ of 0.38 g cm^-3^. WD_branch_ ranged between 0.50 and 0.78 g cm^-3^ in angiosperms (**Table 4**), significantly denser than WD_root_ (**Table 3**). In contrast to WD_branch_, species-means of root and stem WD were highly positively correlated (p=0.002, **Figure 5**). WD_stem_ was significantly greater in evergreen compared to deciduous trees (p=0.031, **Table 2**). WDs of roots, stem and branches were significantly negatively correlated to $K_{S:root}^{pot}$ (**Figure 5**). Negative correlations were found between WD_branch_ and $K_{S:branch}^{hydr}$ , as well as between WD_stem_ and $K_{S:branch}^{pot}$ , D_h:branch_, and F_branch_.

**Table 4:** Wood tissue density (WD; g cm^-3^) of coarse roots, stem and branches of 14 woody species in a seasonally dry Ethiopian Highland forest; see **Table 1** for details.

Leaf habit	Species	WD_root_	WD_stem_	WD_branch_
**Angiosperm, deciduous**	*A. schimperiana*	0.60 ± 0.012 de	0.49 ± 0.013 ab	0.62 ± 0.040 ab
	*B. micrantha*	0.57 ± 0.018 cde	0.58 ± 0.005 bcd	0.64 ± 0.024 ab
	*C. macrostachyus*	0.39 ± 0.018 ab	0.47 ± 0.011 ab	0.50 ± 0.072 a
	*C. molle*	0.48 ± 0.010 abcd	0.62 ± 0.025 cde	0.67 ± 0.046 ab
	*S. abyssinica*	0.38 ± 0.029 a	0.44 ± 0.013 a	0.74 ± 0.079 b
	Mean	0.48 ± 0.045 A	0.52 ± 0.034 A	0.63 ± 0.039 A
**Angiosperm, evergreen**	*A. dimidiata*	0.53 ± 0.047 bcde	0.57 ± 0.006 bcd	0.63 ± 0.050 ab
	*C. aurea*	0.67 ± 0.064 efa	0.75 efa	0.61 ± 0.053 ab
	*C. mildbraedii*	0.77 ± 0.044 fa	0.82 fa	0.78 ± 0.035 b
	*D. abyssinica*	0.53 ± 0.020 bcde	0.71 ± 0.014 efa	0.64 ± 0.031 ab
	*E. capensis*	0.50 ± 0.022 abcd	0.54 ± 0.015 abc	0.71 ± 0.038 ab
	*M. lanceolata*	0.48 ± 0.036 abcd	0.56 ± 0.010 abc	0.50 ± 0.018 a
	*P. africana*	0.56 ± 0.015 cde	0.68 ± 0.036 defa	0.63 ± 0.019 ab
	*T. nobilis*	0.52 ± 0.024 abcde	n.a.	0.75 ± 0.038 b
	Mean	0.57 ± 0.035 A	0.66 ± 0.038 B	0.66 ± 0.032 A
**Gymnosperm**	*A. falcatus*	0.42 ± 0.002 abc	0.46 ± 0.004 ab	0.48 ± 0.056 a

^n.a.^ not available.

Small letters indicate significant differences between species within each organ, large letters indicate significant differences between means of deciduous/evergreen angiosperms (Tukey test, p<0.05; mean **±** SE; n_roots_=4-6, n_stem_=3-6, n_branch_=5-6).

## 4 Discussion

We conducted measurements of branch, stem, and coarse root traits related to sap transport and mechanical support in 14 tree species within a seasonally dry subtropical highland forest in Ethiopia, an ecosystem that has been undersurveyed in terms of tree hydraulic traits. Our findings revealed that empirically determined xylem-specific conductivity ( $K_{S}^{hydr}$ ) in 2nd-year branches of angiosperms ranged from 1.0 to 6.8 kg m^−1^ MPa^−1^ s^-1^, which is similar to previous findings in 355 (sub-)tropical trees from other studies (Santiago et al., 2004; Markesteijn et al., 2011; Kotowska et al., 2015; Zhu et al., 2013). Notably, to our knowledge, our measurements of root hydraulic conductivity, ranging from 1.5 to 115 kg m^−1^ MPa^−1^ s^-1^, are novel for seasonally dry (sub-)tropical forests. We observed similar ( $K_{S}^{hydr}$ and root-to-branch ratios between different leaf habit types. Furthermore, we found good correspondence between $K_{S}^{hydr}$ and potential conductivity $K_{S}^{pot}$ (**Supplementary Figure S3**), although we acknowledge potential confounding effects of the measurement technique, such as sample length, and caution should be exercised when making comparisons to other studies.

### 4.1 Hydraulic efficiencies of roots and branches

Our results revealed a strong decline in hydraulic efficiency in most Ethiopian highland tree species, with measured conductivity in coarse roots 5.1- or 5.8-times greater than in similar-sized branches in deciduous and evergreen angiosperms, respectively. Although hydraulic efficiency is largely determined by vessel diameter, the vessel lumen fraction (F) was closely associated with hydraulic conductivity K_S_ in roots and branches. Narrower conduits were (partially) compensated for by higher conduit densities (CD) in branches of a majority of species (Ewers et al., 2007). The results on hydraulic tapering follow patterns already observed by e.g. Maherali et al. (2006), whereas in their study $K_{S}^{hydr}$ of roots was on average approximately 6-times greater than in branches. Similar, Plavcová et al. (2019) recently revealed significantly higher mean xylem cell cross-sectional areas in roots than in branches of temperate trees. Reducing hydraulic efficiency along the hydraulic flow paths has been observed in many temperate (Lübbe et al., 2022), and tropical tree species (Schuldt et al., 2013; Kotowska et al., 2015), but also some woody species in Mediterranean or semi-arid systems (Martínez-Vilalta et al., 2002; Pratt et al., 2007; Longui et al., 2012; Santini et al., 2018). The phenomenon has been related to interrelated gradients of e.g. turgor pressure (Woodruff et al., 2004; Woodruff and Meinzer, 2011) and cambial age (Li et al., 2019; Rodriguez-Zaccaro et al., 2019) along the flow path. The substantial difference of hydraulic efficiency between roots and branches may be explained by a trade-off between hydraulic efficiency and safety against cavitation, whereas roots were thought to be more vulnerable to cavitation and closer to their hydraulic limit (Martínez-Vilalta et al., 2002). However, this view is increasingly challenged (Gleason et al., 2016; Santini et al., 2018). Recently, Lübbe et al. (2022) reported that xylem embolism resistance did not differ significantly between roots and branches in three out of four temperate tree species. However, in contrast to continuous vessel tapering along the hydraulic pathway from roots in branches observed in temperate and semi-arid systems, a hump-shaped vessel diameter variation has been found in mature trees from tropical moist forests. Largest vessel diameters were present in the coarse root and stem xylem, while smaller vessels were found in small roots and branches-the reason for this observation remains speculative (Schuldt et al., 2013; Kotowska et al., 2015). In our data set, species-specific root-to-branch ratios of c. 1 were found between similar-sized roots and branches. For example, hydraulically weighted conduit diameter (D_h_) in roots of *C. mildbraedii* were only marginally greater than in branches (**Figure 2**) and remained largely similar in *E. capensis*, pointing towards species-specific tradeoffs between hydraulic and other (e.g. mechanical) traits. Plavcová et al. (2019) indicated that the mechanical properties of roots and stems within species are rather independent of each other. While Schuldt et al. (2013) suggested that vessel diameter in above- and belowground organs may be related to segment diameter in trees growing in a perhumid tropical environment, a ΔD_h_ of 30-40% between similar-sized root and branches of this study clearly points towards additional effects in the studied highland forest. However, different tissue differentiation (‘secondary growth’) due to a potentially greater age of sampled coarse root segments compared to 2^nd^-year branches cannot be excluded—roots are notoriously hard to classify (Freschet et al., 2021). In sum, our data provides convincing evidence that the coarse root xylem holds a greater hydraulic efficiency than the branch xylem in subtropical tree species of the seasonally dry Ethiopian highlands.

### 4.2 Relationship between root and branch hydraulic traits in drought-deciduous and evergreen species

We had hypothesized that drought-deciduous and evergreen angiosperms differ in their hydraulic traits and have different relationships between root and branch hydraulic traits. Deciduous species invest less mass per unit leaf area while evergreen species have higher leaf mass per area, constituting different costs and benefits of leaf construction (Eamus, 1999; Powers and Tiffin, 2010; Souza et al., 2020). Both leaf habits often differ in physiological leaf traits (Mediavilla and Escudero, 2003; Ishida et al., 2006) and overall growth strategies (Tomlinson et al., 2014). Recently, however, Ribeiro et al. (2022) found no difference in leaf traits between evergreens and deciduous species in neotropical dry forests. As leaf shedding in seasonally dry environments may present an relative effective mechanism (‘hydraulic fuse’) to prevent or limit damage to a hydraulically vulnerable xylem and overall plant desiccation (Wolfe et al., 2016), we expected that different leaf habits may go along with different hydraulic traits along the flow path. In accordance with (Choat et al., 2005, for branches), our anatomical comparison showed significant greater D_h_ in branches of drought-deciduous trees and tendencies towards greater conduit density (i.e. ‘hydraulic redundancy’) in evergreens’ roots and branches. While Hoeber et al. (2014) did not find significant differences between root and branch traits of leaf habit groups in the seasonally dry tropics of Costa Rica, their trees were only c. 6 years old and large changes in hydraulic strategies have been reported with ongoing tree maturation (Osazuwa-Peters et al., 2017). However, while we hypothesized differences in root-to-branch ratios between leaf habits, i.a. greater root-to-branch ratios in evergreen species, no such differences were observed. This indicates that drought-deciduousness does not translate into a less marked difference between root and branch hydraulic efficiencies compared to evergreen angiosperms. While one could then speculate on a tight coordination between coarse roots and branch traits, this is not supported by our data—which indicated rather weak correlations of hydraulic traits between lateral woody organs. Instead, the higher values of potential K_S_ and leaf area-specific conductivity in deciduous species may allow for an increased carbon gain, and the lower wood density of stems of deciduous trees would enable a high volumetric growth during a shortened growing season (Eamus and Prior, 2001). While Markesteijn et al. (2011) pointed out that drought sensitivity in terms of critical xylem tension is not necessarily lower in deciduous species, Fu et al. (2012) reported greater embolism resistance in evergreen species of Asian tropical dry forests.

While the absence of vulnerability measurements does not allow clarifying this for the studied species, our findings demonstrate that deciduous and evergreen species show marked differences in hydraulic strategies but also highlight a great variability within leaf habit groups, similar to earlier findings in moist tropical forests (Kraft et al., 2008; Hoeber et al., 2014). Here, potential reasons may lay in the unstable precipitation patters in the region, where global climatic events such as El Niño can cause severe rainfall deficits in the Ethiopian summer (Gleixner et al., 2017), potentially resulting in year-to-year differences in most successful hydraulic strategies. Additional traits such as rooting depth (Hasselquist et al., 2010), canopy size and leaf-to-sapwood area (Mencuccini et al., 2019; Rosas et al., 2019), and particular additional leaf traits (e.g., degree of isohydry; (Hartmann et al., 2021); length of leave-less periods (Borchert et al., 2002)), are needed to further unravel species-specific patterns.

### 4.3 Hydraulic efficiency as related to wood density

Wood density (WD) is a key plant property, related to mechanical and (eco-)physiological performance (Martínez-Cabrera et al., 2009; Ziemińska et al., 2013). The relationship between wood density and the underlying anatomical traits is shaped by multiple, interrelated functions of xylem—water transport, storage, and mechanical support (Chave et al., 2009; Messier et al., 2017). A strong negative relationship of WD with lumen fraction of stems and partially across organs has e.g. been demonstrated for different temperate and Mediterranean taxa (Preston et al., 2006; Zanne et al., 2010; Lübbe et al., 2022). This trade-off is a corner-stone of the wood economic spectrum, where denser wood is expected to provide better mechanical support while typically involving restrained sap transport capacities (Messier et al., 2017). However, studies on tropical trees did not find a close linkage between WD_stem_ and vessel characteristics (Fan et al., 2012; Schuldt et al., 2013; Hietz et al., 2017), because WD_stem_ is more controlled by fiber than by conduit structure (Martínez-Cabrera et al., 2009; Ziemińska et al., 2013). Similar, Fortunel et al. (2014) found no relationship between WD and vascular traits at the branch level, suggesting no tradeoff between WD_branch_ and hydraulic efficiency in trees of the moist tropics. In accordance, our pairwise correlation analyses revealed that WD_branch_ had a strong negative relationship with empirically determined $K_{S}^{hydr}$ , but no significant correlations with traits related to either conduit size or frequency. In contrast, angiosperm roots held strong negative relationship between WD_root_ and K_S_, mediated through the negative effect of WD_root_ on D_h_ and lumen fraction F. The higher variation in F_root_ as compared to F_branch_ might explain the good correlation with WD_root_ (R² = 0.61). The correlation between F_branch_ and WD_branch_ revealed a lower goodness-of-fit, with R² being 0.11. WD_root_ of trees in the studied ecosystem may thus to be both determined and restricted by the cumulative conduit volume in the stele, although the scatterplots illustrate the large dependency of WD_root_ on D_h_ and not conduit density in angiosperms. In contrast to our hypothesis, the hydraulic efficiency of coarse roots can thus be predicted reasonably well from WD due its large dependency on conduit properties. WD_root_ of the studied species may thus not depend strongly on fiber properties, as reported for Mediterranean, temperate and tropical tree species (Martínez-Cabrera et al., 2009; Schuldt et al., 2013; Fortunel et al., 2014; Plavcová et al., 2019).

Earlier, Nakagawa et al. (2016) reported that root wood traits were highly correlated with corresponding stem wood traits in South-East Asian tropical trees. Based on these findings, we had hypothesized that WD of coarse roots, stems and branches are generally related. Indeed, our analyses showed that WD_stem_ was highly negatively correlated to root hydraulic conductivity and significantly positively to WD_root_. However, while Fortunel et al. (2014) reported that WD was strongly correlated between branches and roots in 113 Amazonian rainforest tree species across a large environmental gradient, we found no correlations between root and branch WD or between WD_branch_ and WD_stem_. In contrast to our hypothesis, our result thus indicate fundamental differences between above- and belowground xylem: fibers may play a greater role in shaping WD in branches, likely because of their role for mechanical support, while vessels may play a greater role in shaping WD_root_ where the soil matrix provides mechanical support (Pratt et al., 2007). However, as the arrangement of xylem traits and the total fraction of tissues (i.e. fiber wall fraction/lumen fraction, fraction of parenchyma) can also influence wood density (e.g. Ziemińska et al., 2013; Lachenbruch and McCulloh, 2014), this has to be considered in future studies.

## 5 Conclusions

Hydraulic traits and their anatomical bases are crucial for growth and survival and therefore essential to understand species performance and life history strategies. This study, carried out on mature trees of 14 species in a seasonally dry (sub-)tropical forest of the Ethiopian highlands, advances our understanding of root anatomy and hydraulic strategies by confirming that coarse roots exhibit larger xylem vessel diameters and lower wood densities than similar-sized branches of the same species. Differences in hydraulic efficiencies can be as large or even larger between roots and branches compared to species-specific differences. Greater hydraulic efficiency mediated by greater D_h_, was found in branches of drought-deciduous angiosperms compared to evergreen species. However, the differentiation of species based on discrete leaf habits may obscure key information—as the variability of hydraulic traits within leaf habit groups was large. In sum, our study opens new research avenues for a more mechanistic understanding of ecosystem functioning and species assemblages in seasonally dry tropical forests.

## Data availability statement

The raw data supporting the conclusions of this article is available: https://doi.org/10.5281/zenodo.7919399.

## Author contributions

BR, PH, and MS designed the experiments; MS performed the fieldwork and measurements; MS analyzed the data with help of BR; MS prepared the illustrations; MS and BR prepared the first draft, PH and BS revised, and all authors jointly edited the final manuscript. All authors contributed to the article and approved the submitted version.

## References

[B1] Abebe G. (2017). Long-term climate data description in Ethiopia. Data Brief 14, 371–392. doi: 10.1016/j.dib.2017.07.052 28831403 PMC5552378

[B2] Aerts R. van Overtveld K. November E. Wassie A. Abiyu A. Demissew S.. (2016). Conservation of the Ethiopian church forests: threats, opportunities and implications for their management. Sci. Total Environ. 551-552, 404–414. doi: 10.1016/j.scitotenv.2016.02.034 26881731

[B3] Anderegg W. R. L. Klein T. Bartlett M. Sack L. Pellegrini A. F. A. Choat B.. (2016). Meta-analysis reveals that hydraulic traits explain cross-species patterns of drought-induced tree mortality across the globe. Proc. Natl. Acad. Sci. U.S.A. 113, 5024–5029. doi: 10.1073/pnas.1525678113 27091965 PMC4983847

[B4] Anfodillo T. Petit G. Crivellaro A. (2013). Axial conduit widening in woody species: a still neglected anatomical pattern. IAWA J. 34, 352–364. doi: 10.1163/22941932-00000030

[B5] Assefa D. Godbold D. L. Belay B. Abiyu A. Rewald B. (2018). Fine root morphology, biochemistry and litter quality indices of fast- and slow-growing woody species in Ethiopian highland forest. Ecosystems 21, 482–494. doi: 10.1007/s10021-017-0163-7

[B6] Assefa D. Mentler A. Sandén H. Rewald B. Godbold D. L. (2022). The biological origins of soil organic matter in different land-uses in the highlands of Ethiopia. Forests 13, 560. doi: 10.3390/f13040560

[B7] Assefa D. Rewald B. Sandén H. Rosinger C. Abiyu A. Yitaferu B.. (2017). Deforestation and land use strongly effect soil organic carbon and nitrogen stock in Northwest Ethiopia. CATENA 153, 89–99. doi: 10.1016/j.catena.2017.02.003

[B8] Bates D. Mächler M. Bolker B. Walker S. (2015). Fitting linear mixed-effects models using lme4. J. Stat. Soft. 67. doi: 10.18637/jss.v067.i01

[B9] Belay B. Pötzelsberger E. Sisay K. Assefa D. Hasenauer H. (2018). The carbon dynamics of dry tropical afromontane forest ecosystems in the amhara region of Ethiopia. Forests 9, 18. doi: 10.3390/f9010018

[B10] Bingham M. G. Willemen A. Wursten B. T. Ballings P. Hyde M. A. (2021). Flora of Zimbabwe. Accessed June 15, 2022. https://www.zambiaflora.com/index.php.

[B11] Borchert R. Rivera G. Hagnauer W. (2002). Modification of vegetative phenology in a tropical semi-deciduous forest by abnormal drought and rain. Biotropica 34, 27–39. doi: 10.1111/j.1744-7429.2002.tb00239.x

[B12] Chave J. Coomes D. Jansen S. Lewis S. L. Swenson N. G. Zanne A. E. (2009). Towards a worldwide wood economics spectrum. Ecol. Lett. 12, 351–366. doi: 10.1111/j.1461-0248.2009.01285.x 19243406

[B13] Chidumayo E. N. (2005). Effects of climate on the growth of exotic and indigenous trees in central Zambia. J. Biogeography 32, 111–120. doi: 10.1111/j.1365-2699.2004.01130.x

[B14] Choat B. Ball M. C. Luly J. G. Holtum J. A. M. (2005). Hydraulic architecture of deciduous and evergreen dry rainforest tree species from north-eastern Australia. Trees 19, 305–311. doi: 10.1007/s00468-004-0392-1

[B15] Dressler S. Schmidt M. Zizka G. (2011). Central African plants - A Photo Guide. Accessed June 15, 2022. www.centralafricanplants.org.

[B16] Eamus D. (1999). Ecophysiological traits of deciduous and evergreen woody species in the seasonally dry tropics. Trends Ecol. Evol. 14, 11–16. doi: 10.1016/S0169-5347(98)01532-8 10234241

[B17] Eamus D. Prior L. (2001). Ecophysiology of trees of seasonally dry tropics: comparisons among phenologies. Adv. Ecol. Res. 32, 113–197. doi: 10.1016/S0065-2504(01)32012-3

[B18] Eissenstat D. M. Yanai R. D. eds. (2002). Plant Roots: Root Life Span, Efficiency, and Turnover. Boca Raton: CRC Press.

[B19] Eshete A. W. (2007). Ethiopian Church forests: opportunities and challenges for restoration (Wageningen, The Netherlands: Wageningen University and Research, Forest Ecology and Forest Management Group, Centre for Ecosystem Studies).

[B20] Ewers F. W. Ewers J. M. Jacobsen A. L. López-Portillo J. (2007). Vessel redundancy: modeling safety in numbers. IAWA J. 28, 373–388. doi: 10.1163/22941932-90001650

[B21] Fan Z.-X. Zhang S.-B. Hao G.-Y. Ferry Slik J. W. Cao K.-F. (2012). Hydraulic conductivity traits predict growth rates and adult stature of 40 Asian tropical tree species better than wood density. J. Ecol. 100, 732–741. doi: 10.1111/j.1365-2745.2011.01939.x

[B22] Fortunel C. Ruelle J. Beauchêne J. Fine P. V. A. Baraloto C. (2014). Wood specific gravity and anatomy of branches and roots in 113 Amazonian rainforest tree species across environmental gradients. New Phytol. 202, 79–94. doi: 10.1111/nph.12632 24329812

[B23] Freschet G. T. Pagès L. Iversen C. M. Comas L. H. Rewald B. Roumet C.. (2021). A starting guide to root ecology: strengthening ecological concepts and standardising root classification, sampling, processing and trait measurements. New Phytol. 232, 973–1122. doi: 10.1111/nph.17572 34608637 PMC8518129

[B24] Fu P.-L. Jiang Y.-J. Wang A.-Y. Brodribb T. J. Zhang J.-L. Zhu S.-D.. (2012). Stem hydraulic traits and leaf water-stress tolerance are co-ordinated with the leaf phenology of angiosperm trees in an Asian tropical dry karst forest. Ann. Bot. 110, 189–199. doi: 10.1093/aob/mcs092 22585930 PMC3380589

[B25] Fulton M. R. Kamman J. C. Coyle M. P. (2014). Hydraulic limitation on maximum height of *Pinus strobus* trees in northern Minnesota, USA. Trees 28, 841–848. doi: 10.1007/s00468-014-0996-z

[B26] Gleason S. M. Butler D. W. Ziemińska K. Waryszak P. Westoby M. (2012). Stem xylem conductivity is key to plant water balance across Australian angiosperm species. Funct. Ecol. 26, 343–352. doi: 10.1111/j.1365-2435.2012.01962.x

[B27] Gleason S. M. Westoby M. Jansen S. Choat B. Hacke U. G. Pratt R. B.. (2016). Weak tradeoff between xylem safety and xylem-specific hydraulic efficiency across the world's woody plant species. New Phytol. 209, 123–136. doi: 10.1111/nph.13646 26378984

[B28] Gleixner S. Keenlyside N. Viste E. Korecha D. (2017). The El niño effect on Ethiopian summer rainfall. Clim Dyn 49, 1865–1883. doi: 10.1007/s00382-016-3421-z

[B29] Hacke U. G. Sperry J. S. (2001). Functional and ecological xylem anatomy. Perspect. Plant Ecology Evol. Systematics 4, 97–115. doi: 10.1078/1433-8319-00017

[B30] Hartmann H. Link R. M. Schuldt B. (2021). A whole-plant perspective of isohydry: stem-level support for leaf-level plant water regulation. Tree Physiol. 41, 901–905. doi: 10.1093/treephys/tpab011 33594416 PMC8827077

[B31] Hasselquist N. J. Allen M. F. Santiago L. S. (2010). Water relations of evergreen and drought-deciduous trees along a seasonally dry tropical forest chronosequence. Oecologia 164, 881–890. doi: 10.1007/s00442-010-1725-y 20658152 PMC2981736

[B32] Hietz P. Rosner S. Hietz-Seifert U. Wright S. J. (2017). Wood traits related to size and life history of trees in a Panamanian rainforest. New Phytol. 213, 170–180. doi: 10.1111/nph.14123 27533709

[B33] Hoeber S. Leuschner C. Köhler L. Arias-Aguilar D. Schuldt B. (2014). The importance of hydraulic conductivity and wood density to growth performance in eight tree species from a tropical semi-dry climate. For. Ecol. Manage. 330, 126–136. doi: 10.1016/j.foreco.2014.06.039

[B34] Hothorn T. Bretz F. Westfall P. (2008). Simultaneous inference in general parametric models. Biom J. 50, 346–363. doi: 10.1002/bimj.200810425 18481363

[B35] Hyde M. Wursten B. Petra B. Coates Palgrave M. (2022). Flora of Zimbabwe. Accessed June 15, 2022. https://www.zimbabweflora.co.zw/index.php.

[B36] Ishida A. Diloksumpun S. Ladpala P. Staporn D. Panuthai S. Gamo M.. (2006). Contrasting seasonal leaf habits of canopy trees between tropical dry-deciduous and evergreen forests in Thailand. Tree Physiol. 26, 643–656. doi: 10.1093/treephys/26.5.643 16452078

[B37] Khan A. Sun J. Zarif N. Khan K. Jamil M. A. Yang L.. (2020). Effects of increased n deposition on leaf functional traits of four contrasting tree species in northeast China. Plants 9. doi: 10.3390/plants9091231 PMC7570078 32962033

[B38] Koçillari L. Olson M. E. Suweis S. Rocha R. P. Lovison A. Cardin F.. (2021). The widened pipe model of plant hydraulic evolution. Proc. Natl. Acad. Sci. U.S.A. 118. doi: 10.1073/pnas.2100314118 PMC8179198 34039710

[B39] Kotowska M. M. Hertel D. Rajab Y. A. Barus H. Schuldt B. (2015). Patterns in hydraulic architecture from roots to branches in six tropical tree species from cacao agroforestry and their relation to wood density and stem growth. Front. Plant Sci. 6. doi: 10.3389/fpls.2015.00191 PMC4379754 25873922

[B40] Kraft N. J. B. Valencia R. Ackerly D. D. (2008). Functional traits and niche-based tree community assembly in an Amazonian forest. Science 322, 580–582. doi: 10.1126/science.1160662 18948539

[B41] Kröber W. Zhang S. Ehmig M. Bruelheide H. (2014). Linking xylem hydraulic conductivity and vulnerability to the leaf economics spectrum - a cross-species study of 39 evergreen and deciduous broadleaved subtropical tree species. PloS One 9, e109211. doi: 10.1371/journal.pone.0109211 25423316 PMC4244042

[B42] Kuznetsova A. Brockhoff P. B. Christensen R. H. B. (2017). lmerTest package: tests in linear mixed effects models. J. Stat. Soft. 82, 1–26. doi: 10.18637/jss.v082.i13

[B43] Lachenbruch B. McCulloh K. A. (2014). Traits, properties, and performance: how woody plants combine hydraulic and mechanical functions in a cell, tissue, or whole plant. New Phytol. 204, 747–764. doi: 10.1111/nph.13035 25250668

[B44] Lenth R. V. (2022) Emmeans: estimated marginal means, aka least-squares means. Available at: https://CRAN.R-project.org/package=emmeans (Accessed November 10, 2022).

[B45] Li S. Li X. Link R. Li R. Deng L. Schuldt B.. (2019). Influence of cambial age and axial height on the spatial patterns of xylem traits in *Catalpa bungei*, a ring-porous tree species native to China. Forests 10, 662. doi: 10.3390/f10080662

[B46] Link R. M. (2020) Corrmorant: flexible correlation matrices based on ‘ggplot2’. Available at: http://github.com/r-link/corrmorant (Accessed October 10, 2022).

[B47] Lintunen A. Kalliokoski T. (2010). The effect of tree architecture on conduit diameter and frequency from small distal roots to branch tips in *Betula pendula, picea abies* and *Pinus sylvestris* . Tree Physiol. 30, 1433–1447. doi: 10.1093/treephys/tpq085 21030407

[B48] Longui E. L. Rajput K. S. Galvão de Melo A. C. de Araújo Alves L. do Nascimento C. B. (2017). Root to branch wood anatomical variation and its influence on hydraulic conductivity in five Brazilian cerrado species. Bosque 38, 183–193. doi: 10.4067/S0717-92002017000100018

[B49] Longui E. L. Silva R. d. B. Romeiro D. de Lima I. L. Florsheim S. M. B.. (2012). Root-branch anatomical investigation of *Eriotheca gracilipes* young trees: a biomechanical and ecological approach. Scientia Forestalis 40, 23–33.

[B50] Lübbe T. Lamarque L. J. Delzon S. Torres Ruiz J. M. Burlett R. Leuschner C.. (2022). High variation in hydraulic efficiency but not xylem safety between roots and branches in four temperate broad-leaved tree species. Funct. Ecol. 36, 699–712. doi: 10.1111/1365-2435.13975

[B51] Machado S. R. Rodella R. A. Angyalossy V. Marcati C. R. (2007). Structural variations in root and stem wood of *Styrax* (Styracaceae) from Brazilian forest and cerrado. IAWA J. 28, 173–188. doi: 10.1163/22941932-90001632

[B52] Maherali H. Moura C. E. Caldeira M. C. Willson C. J. Jackson R. B. (2006). Functional coordination between leaf gas exchange and vulnerability to xylem cavitation in temperate forest trees. Plant Cell Environ. 29, 571–583. doi: 10.1111/j.1365-3040.2005.01433.x 17080608

[B53] Markesteijn L. Poorter L. Bongers F. Paz H. Sack L. (2011). Hydraulics and life history of tropical dry forest tree species: coordination of species' drought and shade tolerance. New Phytol. 191, 480–495. doi: 10.1111/j.1469-8137.2011.03708.x 21477008

[B54] Martínez-Cabrera H. I. Jones C. S. Espino S. Schenk H. J. (2009). Wood anatomy and wood density in shrubs: responses to varying aridity along transcontinental transects. Am. J. Bot. 96, 1388–1398. doi: 10.3732/ajb.0800237 21628286

[B55] Martínez-Vilalta J. Prat E. Oliveras I. Piñol J. (2002). Xylem hydraulic properties of roots and stems of nine Mediterranean woody species. Oecologia 133, 19–29. doi: 10.1007/s00442-002-1009-2 24599365

[B56] Martin-StPaul N. K. Longepierre D. Huc R. Delzon S. Burlett R. Joffre R.. (2014). How reliable are methods to assess xylem vulnerability to cavitation? the issue of 'open vessel' artifact in oaks. Tree Physiol. 34, 894–905. doi: 10.1093/treephys/tpu059 25074860

[B57] Mediavilla S. Escudero A. (2003). Stomatal responses to drought at a Mediterranean site: a comparative study of co-occurring woody species differing in leaf longevity. Tree Physiol. 23, 987–996. doi: 10.1093/treephys/23.14.987 12952785

[B58] Mencuccini M. Hölttä T. Petit G. Magnani F. (2007). Sanio's laws revisited. size-dependent changes in the xylem architecture of trees. Ecol. Lett. 10, 1084–1093. doi: 10.1111/j.1461-0248.2007.01104.x 17850336

[B59] Mencuccini M. Rosas T. Rowland L. Choat B. Cornelissen H. Jansen S.. (2019). Leaf economics and plant hydraulics drive leaf : wood area ratios. New Phytol. 224, 1544–1556. doi: 10.1111/nph.15998 31215647

[B60] Méndez-Alonzo R. Paz H. Cruz Zuluaga R. Rosell J. A. Olson M. E. (2012). Coordinated evolution of leaf and stem economics in tropical dry forest trees. Ecology 93, 2397–2406. doi: 10.1890/11-1213.1 23236911

[B61] Messier J. Lechowicz M. J. McGill B. J. Violle C. Enquist B. J. (2017). Interspecific integration of trait dimensions at local scales: the plant phenotype as an integrated network. J. Ecol. 105, 1775–1790. doi: 10.1111/1365-2745.12755

[B62] Nakagawa M. Hori M. Umemura M. Ishida T. (2016). Relationships of wood density and wood chemical traits between stems and coarse roots across 53 bornean tropical tree species. J. Trop. Ecol. 32, 175–178. doi: 10.1017/S0266467416000018

[B63] Olson M. E. Anfodillo T. Gleason S. M. McCulloh K. A. (2021). Tip-to-base xylem conduit widening as an adaptation: causes, consequences, and empirical priorities. New Phytol. 229, 1877–1893. doi: 10.1111/nph.16961 32984967

[B64] Osazuwa-Peters O. L. Wright S. J. Zanne A. E. (2017). Linking wood traits to vital rates in tropical rainforest trees: insights from comparing sapling and adult wood. Am. J. Bot. 104, 1464–1473. doi: 10.3732/ajb.1700242 29885221

[B65] Peel M. C. Finlayson B. L. McMahon T. A. (2007). Updated world map of the köppen-Geiger climate classification. Hydrol. Earth Syst. Sci. 11, 1633–1644. doi: 10.5194/hess-11-1633-2007

[B66] Plavcová L. Gallenmüller F. Morris H. Khatamirad M. Jansen S. Speck T. (2019). Mechanical properties and structure-function trade-offs in secondary xylem of young roots and stems. J. Exp. Bot. 70, 3679–3691. doi: 10.1093/jxb/erz286 31301134

[B67] Poorter L. McDonald I. Alarcón A. Fichtler E. Licona J.-C. Peña-Claros M.. (2010). The importance of wood traits and hydraulic conductance for the performance and life history strategies of 42 rainforest tree species. New Phytol. 185, 481–492. doi: 10.1111/j.1469-8137.2009.03092.x 19925555

[B68] Powers J. S. Tiffin P. (2010). Plant functional type classifications in tropical dry forests in Costa Rica: leaf habit versus taxonomic approaches. Funct. Ecol. 24, 927–936. doi: 10.1111/j.1365-2435.2010.01701.x

[B69] Pratt R. B. Jacobsen A. L. Ewers F. W. Davis S. D. (2007). Relationships among xylem transport, biomechanics and storage in stems and roots of nine rhamnaceae species of the California chaparral. New Phytol. 174, 787–798. doi: 10.1111/j.1469-8137.2007.02061.x 17504462

[B70] Preston K. A. Cornwell W. K. Denoyer J. L. (2006). Wood density and vessel traits as distinct correlates of ecological strategy in 51 California coast range angiosperms. New Phytol. 170, 807–818. doi: 10.1111/j.1469-8137.2006.01712.x 16684240

[B71] R Core Team (2019) R: a language and environment for statistical computing (Vienna, Austria). Available at: https://www.R-project.org/ (Accessed November 17, 2022).

[B72] Reich P. B. (2014). The world-wide ‘fast-slow’ plant economics spectrum: a traits manifesto. J. Ecol. 102, 275–301. doi: 10.1111/1365-2745.12211

[B73] Rewald B. Meinen C. Trockenbrodt M. Ephrath J. E. Rachmilevitch S. (2012). Root taxa identification in plant mixtures – current techniques and future challenges. Plant Soil 359, 165–182. doi: 10.1007/s11104-012-1164-0

[B74] Ribeiro D. R. Silva J. L. A. do Nascimento M. T. Vitória A. P. (2022). Leaf habits and their relationship with leaf and wood traits in tropical dry forests. Trees 36, 7–24. doi: 10.1007/s00468-021-02200-0

[B75] Rodriguez-Zaccaro F. D. Valdovinos-Ayala J. Percolla M. I. Venturas M. D. Pratt R. B. Jacobsen A. L. (2019). Wood structure and function change with maturity: age of the vascular cambium is associated with xylem changes in current-year growth. Plant Cell Environ. 42, 1816–1831. doi: 10.1111/pce.13528 30707440

[B76] Rosas T. Mencuccini M. Barba J. Cochard H. Saura-Mas S. Martínez-Vilalta J. (2019). Adjustments and coordination of hydraulic, leaf and stem traits along a water availability gradient. New Phytol. 223, 632–646. doi: 10.1111/nph.15684 30636323

[B77] RStudio Team (2016) RStudio: integrated development environment for r (Boston, MA). Available at: http://www.rstudio.com/ (Accessed November 17, 2022).

[B78] Santiago L. S. Goldstein G. Meinzer F. C. Fisher J. B. Machado K. Woodruff D. (2004). Leaf photosynthetic traits scale with hydraulic conductivity and wood density in Panamanian forest canopy trees. Oecologia 140, 543–550. doi: 10.1007/s00442-004-1624-1 15232729

[B79] Santini N. S. Cleverly J. Faux R. McBean K. Nolan R. Eamus D. (2018). Root xylem characteristics and hydraulic strategies of species co-occurring in semi-arid Australia. IAWA J. 39, 43–62. doi: 10.1163/22941932-20170188

[B80] Schuldt B. Leuschner C. Brock N. Horna V. (2013). Changes in wood density, wood anatomy and hydraulic properties of the xylem along the root-to-shoot flow path in tropical rainforest trees. Tree Physiol. 33, 161–174. doi: 10.1093/treephys/tps122 23292668

[B81] Schumann K. Leuschner C. Schuldt B. (2019). Xylem hydraulic safety and efficiency in relation to leaf and wood traits in three temperate *Acer* species differing in habitat preferences. Trees 33, 1475–1490. doi: 10.1007/s00468-019-01874-x

[B82] Seyoum Y. Fetene M. Strobl S. Beck E. (2012). Foliage dynamics, leaf traits, and growth of coexisting evergreen and deciduous trees in a tropical montane forest in Ethiopia. Trees 26, 1495–1512. doi: 10.1007/s00468-012-0723-6

[B83] Sisay K. Thurnher C. Belay B. Lindner G. Hasenauer H. (2017). Volume and carbon estimates for the forest area of the amhara region in northwestern Ethiopia. Forests 8, 122. doi: 10.3390/f8040122

[B84] Skelton R. P. Anderegg L. D. L. Lamarque L. J. (2019). Examining variation in hydraulic and resource acquisition traits along climatic gradients tests our understanding of plant form and function. New Phytol. 223, 505–507. doi: 10.1111/nph.15893 31125111

[B85] Souza B. C. Carvalho E. C. D. Oliveira R. S. de Araujo F. S. de Lima A. L. A. Rodal M. J. N. (2020). Drought response strategies of deciduous and evergreen woody species in a seasonally dry neotropical forest. Oecologia 194, 221–236. doi: 10.1007/s00442-020-04760-3 32965523

[B86] Sperry J. S. Donnelly J. R. Tyree M. T. (1988). A method for measuring hydraulic conductivity and embolism in xylem. Plant Cell Environ. 11, 35–40. doi: 10.1111/j.1365-3040.1988.tb01774.x

[B87] Spinoni J. Barbosa P. de Jager A. McCormick N. Naumann G. Vogt J. V.. (2019). A new global database of meteorological drought events from 1951 to 2016. J. Hydrol Reg. Stud. 22, 100593. doi: 10.1016/j.ejrh.2019.100593 32257820 PMC7099764

[B88] Sterck F. J. Zweifel R. Sass-Klaassen U. Chowdhury Q. (2008). Persisting soil drought reduces leaf specific conductivity in scots pine (*Pinus sylvestris*) and pubescent oak (*Quercus pubescens*). Tree Physiol. 28, 529–536. doi: 10.1093/treephys/28.4.529 18244940

[B89] Tilney P. M. Nel M. van Wyk A. E. (2018). Foliar secretory structures in *Ekebergia capensis* (Meliaceae). Heliyon 4, e00541. doi: 10.1016/j.heliyon.2018.e00541 29527579 PMC5842367

[B90] Tomlinson K. W. Poorter L. Bongers F. Borghetti F. Jacobs L. van Langevelde F. (2014). Relative growth rate variation of evergreen and deciduous savanna tree species is driven by different traits. Ann. Bot. 114, 315–324. doi: 10.1093/aob/mcu107 24958787 PMC4111386

[B91] Tyree M. T. Ewers F. W. (1991). The hydraulic architecture of trees and other woody plants. New Phytol. 119, 345–360. doi: 10.1111/j.1469-8137.1991.tb00035.x

[B92] Vinya R. Malhi Y. Brown N. Fisher J. B. (2012). Functional coordination between branch hydraulic properties and leaf functional traits in miombo woodlands: implications for water stress management and species habitat preference. Acta Physiol. Plant 34, 1701–1710. doi: 10.1007/s11738-012-0965-3

[B93] Wassie A. Sterck F. J. Bongers F. (2010). Species and structural diversity of church forests in a fragmented Ethiopian highland landscape. J. Vegetation Sci. 21, 938–948. doi: 10.1111/j.1654-1103.2010.01202.x

[B94] Wassie A. Teketay D. Powell N. (2005). Church Forests in north gonder administrative zone, northern Ethiopia. Forests Trees livelihoods 15, 349–373. doi: 10.1080/14728028.2005.9752536

[B95] Weemstra M. Kuyper T. W. Sterck F. J. Umaña M. N. (2022). Incorporating belowground traits: avenues towards a whole-tree perspective on performance. Oikos 4, 685. doi: 10.1111/oik.08827

[B96] Wolfe B. T. Sperry J. S. Kursar T. A. (2016). Does leaf shedding protect stems from cavitation during seasonal droughts? a test of the hydraulic fuse hypothesis. New Phytol. 212, 1007–1018. doi: 10.1111/nph.14087 27373446

[B97] Woodruff D. R. Bond B. J. Meinzer F. C. (2004). Does turgor limit growth in tall trees? Plant Cell Environ. 27, 229–236. doi: 10.1111/j.1365-3040.2003.01141.x

[B98] Woodruff D. R. Meinzer F. C. (2011). “Size-dependent changes in biophysical control of tree growth: the role of turgor,” in Size- and age-related changes in tree structure and function. Eds. Meinzer F. C. Lachenbruch B. Dawson T. E. (Dordrecht: Springer Netherlands), 363–384.

[B99] Worbes M. Blanchart S. Fichtler E. (2013). Relations between water balance, wood traits and phenological behavior of tree species from a tropical dry forest in Costa Rica–a multifactorial study. Tree Physiol. 33, 527–536. doi: 10.1093/treephys/tpt028 23629581

[B100] Ye Z.-H. (2002). Vascular tissue differentiation and pattern formation in plants. Annu. Rev. Plant Biol. 53, 183–202. doi: 10.1146/annurev.arplant.53.100301.135245 12221972

[B101] Zanne A. E. Westoby M. Falster D. S. Ackerly D. D. Loarie S. R. Arnold S. E. J.. (2010). Angiosperm wood structure: global patterns in vessel anatomy and their relation to wood density and potential conductivity. Am. J. Bot. 97, 207–215. doi: 10.3732/ajb.0900178 21622380

[B102] Zhu S.-D. Song J.-J. Li R.-H. Ye Q. (2013). Plant hydraulics and photosynthesis of 34 woody species from different successional stages of subtropical forests. Plant Cell Environ 36, 879–891. doi: 10.1111/pce.12024 23057774

[B103] Ziemińska K. Butler D. W. Gleason S. M. Wright I. J. Westoby M. (2013). Fibre wall and lumen fractions drive wood density variation across 24 Australian angiosperms. AoB Plants 5, 25. doi: 10.1093/aobpla/plt046

