# Supplementary material for: Root and branch hydraulic functioning and trait coordination across organs in drought-deciduous and evergreen tree species of a subtropical highland forest
Source: Front Plant Sci. 2023 Jun 12;14:1127292. doi: 10.3389/fpls.2023.1127292 (PMC10291250; doi:10.3389/fpls.2023.1127292)
Supplement: Supplementary file 1 [file DataSheet_1.pdf]

Supplementary Material

Root and branch hydraulic functioning and trait coordination across organs in drought-deciduous and evergreen tree species of a subtropical highland forest

Marian Schönauer, Peter Hietz, Bernhard Schuldt, Boris Rewald*

*** Correspondence:** Boris Rewald: brewald@rootecology.de

1 Supplementary Methods for K_L

Leaf area-specific conductivity (K_L, kg m^{-1} MPa^{-1} s^{-1}) of branches was calculated as maximum K_h (after flushing) per distal leaf area (m², one-sided) per branch. To calculate the distal leaf area, the specific leaf area (SLA; cm^2 g^{-1}) of 5 leaves per branch was calculated using a flatbed scanner and the PC software WinFolia 2013 Pro (Regent Instruments, Canada) to determine the leaf area, and the respective dry mass (Vile et al.,2005; Woldeamanual, 2016) . The total distal leaf area per branch was calculated by multiplying the SLA per branch with the total distal leaf dry mass (data not shown).

Vile, D., Garnier, E., Shipley, B., Laurent, G., Navas, M.-L., Roumet, C., et al. (2005). Specific leaf area and dry matter content estimate thickness in laminar leaves. Annals of Botany 96, 1129–1136. doi: 10.1093/aob/mci264

Woldeamanual, L.H. (2016). Leaf traits of church forest trees in North West Ethiopia. Master thesis. Vienna: University of Natural Resources and Life Sciences Vienna, Institute of Forest Ecology (IFE).

2 Supplementary Figures and Tables

2.1 Supplementary Tables

Supplementary Table S1. List of anatomical and xylem-hydraulic traits used; with units, definitions and the organ determined on.

Abbreviation[§]	Unit	Trait definition (organ[§])
CD	n mm^{-2}	Conduit density (root, branch)
CL	cm	Maximum conduit length (branch)
D_h	µm	Hydraulically weighted conduit diameter (root, branch)
F		Conduit lumen fraction (root, branch)
WD	g cm^{-3}	Wood density (root, branch, stem)
HV	m^2 m^{-2}	Huber value, i.e. leaf area-specific sapwood area, one-sided (branch)
K_h	kg m MPa^{-1} s^{-1}	Measured axial hydraulic conductivity (root, branch)
K_L	kg m^{-1} MPa^{-1} s^{-1}	Leaf area-specific conductivity, one-sided (branch)
K_S^{hydr}	kg m^{-1} MPa^{-1} s^{-1}	Measured xylem-specific conductivity (root, branch); partially displayed log-transformed
K_S^{pot}	kg m^{-1} MPa^{-1} s^{-1}	Potential xylem-specific conductivity as determined by anatomical analyses (root, branch); partially displayed log-transformed
R:B	-	Root-to-branch ratios of specific traits (root, branch)

[§]Subscripts 'root', 'branch' or 'stem' on abbreviations in the text mark trait values of specific organs

Supplementary Table S2. Diameter (mm) of coarse roots and 2nd-year branches (and their pith area) of 14 woody species of a seasonally dry Ethiopian Highland forest used for measuring xylem-specific hydraulic conductivities K_S^{hydr} (mean±SE; n = 6).

Phylum, leaf habit	Species	Diameter (mm)		
		Coarse root	Branch	Pith (Branch)
**Angiosperm, deciduous**	*A. schimperiana*	4.0±0.3	5.2±0.2	1.6±0.2
	B. micrantha	3.9±0.4	4.8±0.3	1.8±0.2
	C. macrostachyus	3.9±0.4	6.6±0.3	2.7±0.2
	C. molle	4.5±0.5	5.9±0.3	1.7±0.1
	S. abyssinica	2.8±0.3	6.7±0.2	4.0±0.2
**Angiosperm, evergreen**	*A. dimidiata*	3.8±0.3	4.4±0.4	1.4±0.2
	C. aurea	4.5±0.2	5.5±0.3	1.8±0.2
	C. mildbraedii	3.7±0.3	4.2±0.2	1.2±0.1
	D. abyssinica	3.9±0.4	4.7±0.4	1.6±0.3
	E. capensis	3.7±0.3	5.7±0.2	2.8±0.1
	M. lanceolata	4.9±0.5	5.4±0.5	2.1±0.3
	P. africana	3.9±0.3	3.9±0.2	1.0±0.1
	T. nobilis	4.4±0.4	4.7±0.2	1.4±0.1
**Gymnosperm**	*A. falcatus*	4.3±0.6	3.7±0.4	0.6±0.1

Supplementary Table S3. Partial lumen area (F, %) of coarse roots and 2nd-year branches, leaf area-specific conductivity (K_L), and Huber value (HV) of branches in 14 woody species of a seasonally dry Ethiopian Highland forest; see Table 1 for details. Small letters indicate significant differences between species within each parameter, large letters indicate significant differences between means of deciduous/evergreen angiosperms (Tukey test, $p<0.05$; mean±SE; $n_F=3$; $n_{KL/HV}=2\text{-}3$).

Phylum, leaf habit	Species	F_{root}	F_{branch}	K_L	$HV*10^4$
**Angiosperm, deciduous**	*A. schimperiana*	9.5±0.0 ab	10±1.3 a	16000±1600 e	44±7.3 bc
	B. micrantha	13±3.5 ab	15±0.9 ab	900±110 c	3.3±0.21 a
	C. macrostachyus	37±6.1 b	17±2.5 ab	3000±510 d	5.3±0.56 ab
	C. molle	18±1.1 ab	11±1.7 a	1600±140 cd	9.9±0.18 ab
	S. abyssinica	27±3.0 ab	15±3.3 ab	13000±4700 e	70±24 c
	Mean	21±5.0 A	14±1.3 A	6900±3200 A	26±13 A
**Angiosperm, evergreen**	*A. dimidiata*	27±2.1 ab	11 ab	900±77 c	4.1±0.35 ab
	C. aurea	19±3.4 ab	8.3±0.1 a	310±82 b	1.4±0.1 a
	C. mildbraedii	6.2±1.4 a	7.4±1.2 a	820±100 c	5.8±0.6 ab
	D. abyssinica	27±4.8 ab	13±1.5 a	680±140 bc	2.4±0.3 a
	E. capensis	23±2.7 ab	18±1.1 ab	15000±3800 e	80±10 c
	M. lanceolata	29±4.5 ab	16±0.1 ab	950±210 c	3.4±0.55 a
	P. africana	19±3.1 ab	11±1.2 a	940±190 c	3.9±1.2 ab
	T. nobilis	25±1.5 ab	7.5±0.9 a	910±110 c	10±1.6 ab
	Mean	22±2.6 A	11±1.4 A	2600±1800 A	14±9.5 A
**Gymnosperm**	*A. falcatus*	37±0.1 b	26±0.8 b	37±7.3 a	0.7±0.074 a

Supplementary Table S4. Coarse root to branch (R:B) ratios of specific trait values of five drought-deciduous and eight evergreen woody angiosperms and one gymnosperm in a seasonally dry Ethiopian Highland forest. Traits: potential (K_S^{pot}) and hydraulic xylem-specific conductivity (K_S^{hydr}, kg m^{-1} MPa^{-1} s^{-1}), hydraulically weighted conduit diameter (D_h, µm), conduit density (CD, n mm^{-2}), and wood density (WD, g cm^{-3}). Small letters indicate significant differences between species, large letters denote significant differences between means of deciduous / evergreen Angiosperms (Tukey test, $p<0.05$; mean±SE).

Phylum, Leaf habit	Species	Coarse root to branch (R:B) ratios				
		**K_S^{hydr}§**	**K_S^{pot}**	**D_h**	**CD**	**WD§**
**Angiosperm, deciduous**	*A. schimperiana*	n.a.	3.7	1.3	1.4	0.99±0.4 b
	B. micrantha	2.8±1.1 a	4.1	1.6	0.63	0.91±0.37 b
	C. macrostachyus	2.9±1.2 ab	18.0	2.8	0.31	0.85±0.35 b
	C. molle	8.3±3.4 abc	0.5	0.8	1.3	0.73±0.30 ab
	S. abyssinica	6.5±2.7 abc	11.0	2	0.78	0.53±0.22 a
	Mean	5.1±2.1 A	7.5 A	1.7 A	0.88 A	0.80±0.33 A
**Angiosperm, evergreen**	*A. dimidiata*	5.6±2.3 ab	2.4	1.7	0.27	0.85±0.35 ab
	C. aurea	4.1±1.7 ab	4.4	1.7	0.73	1.00±0.41 b
	C. mildbraedii	1.5±0.6 a	5.7	1.6	0.8	1.00±0.41 b
	D. abyssinica	4.4±1.8 ab	4.5	1.4	1.1	0.87±0.36 b
	E. capensis	3.7±1.5 ab	1.4	1.0	1.5	0.72±0.29 ab
	M. lanceolata	5.3±2.2 ab	10.0	2.2	0.42	0.97±0.40 b
	P. africana	8.0±3.3 abc	5.1	1.7	0.54	0.89±0.36 b
	T. nobilis	14.0±5.7 c	6.3	2.3	0.22	0.70±0.29 ab
	Mean	5.8±2.4 A	5.0 A	1.7 A	0.70 A	0.88±0.36 A
**Gymnosperm**	*A. falcatus*	11.0±4.5 bc	1.3	1.3	0.42	0.80±0.33 ab

§Comparisons between individual species could be conducted for the traits K_S^{hydr} and WD only due to replication; n.a. = not available

Supplementary Table S5. Regression coefficients of root and branch traits of 14 woody species (i.e. 'All Species') or angiosperms (excluding the gymnosperm) of an Ethiopian Highland forest. Traits: Wood density (WD, g cm^{-3}), hydraulically weighted conduit diameter (D_h, µm), conduit density (CD, n mm^{-2}), conduit lumen fraction (F, %), potential (K_S^{pot}) and measured xylem-specific conductivity (K_S^{hydr}, kg m^{-1} MPa^{-1} s^{-1}).

Data	Trait	Coefficient	Estimate	Std.Error	t.value	p.value
**All Species**	D_h	(Intercept)	19	16	1.1	0.28
		$D_{h:branch}$	1.1	0.43	2.5	0.026
	CD	(Intercept)	86	18	4.9	0.00039
		CD_{branch}	0.2	0.015	13	1.8e-08
	F	(Intercept)	7.2	5.7	1.3	0.22
		F_{branch}	1.2	0.4	2.9	0.013
	K_S^{pot}	(Intercept)	16	14	1.2	0.26
		$K_{S:branch}^{pot}$	2.8	1.2	2.3	0.04
	K_S^{hydr}	(Intercept)	6.5	2.1	3.2	0.0091
		$K_{S:branch}^{hydr}$	1.6	0.78	2	0.07
	WD	(Intercept)	0.24	0.19	1.2	0.24
		WD_{branch}	0.46	0.3	1.5	0.15
**Angiosperms**	D_h	(Intercept)	24	23	1.1	0.31
		$D_{h:branch}$	0.96	0.57	1.7	0.12
	CD	(Intercept)	48	70	0.69	0.5
		CD_{branch}	0.45	0.44	1	0.33
	F	(Intercept)	6.3	7.8	0.81	0.43
		F_{branch}	1.2	0.61	2	0.066
	K_S^{pot}	(Intercept)	19	16	1.17	0.26
		$K_{S:branch}^{pot}$	2.6	1.3	2.0	0.07
	K_S^{hydr}	(Intercept)	7.3	2.3	3.1	0.01
		$K_{S:branch}^{hydr}$	1.3	0.84	1.6	0.14
	WD	(Intercept)	0.28	0.23	1.2	0.24
		WD_{branch}	0.39	0.35	1.1	0.29

Supplementary Table S6. Regression coefficients of linear mixed-effects models, with vessel diameter (D_h, µm), logarithmic values of empirically determined hydraulic conductivity (K_S^{hydr}, kg m^{-1} MPa^{-1} s^{-1}) of branches and roots as response versus the independent operators' tree height (h, [m]) and random effects of species.

	$D_{h:branch}$		$D_{h:root}$		$log(K_{S:branch}^{hydr})$		$log(K_{S:root}^{hydr})$	
Coefficient	(Intercept)	h	(Intercept)	h	(Intercept)	h	(Intercept)	h
Estimate	40	-0.14	54	0.50	0.75	-0.0045	2.5	-0.046
Std.Error	6.1	0.55	9.9	0.81	0.20	0.015	0.35	0.031
df	15	18	23	21	37	69	24	34
t.value	6.5	-0.26	5.5	0.61	3.8	-0.31	7.2	-1.5
p.value	9.5e-06	0.80	1.40e-05	0.55	6.0e-04	0.76	1.80e-07	0.14

2.2 Supplementary Figures

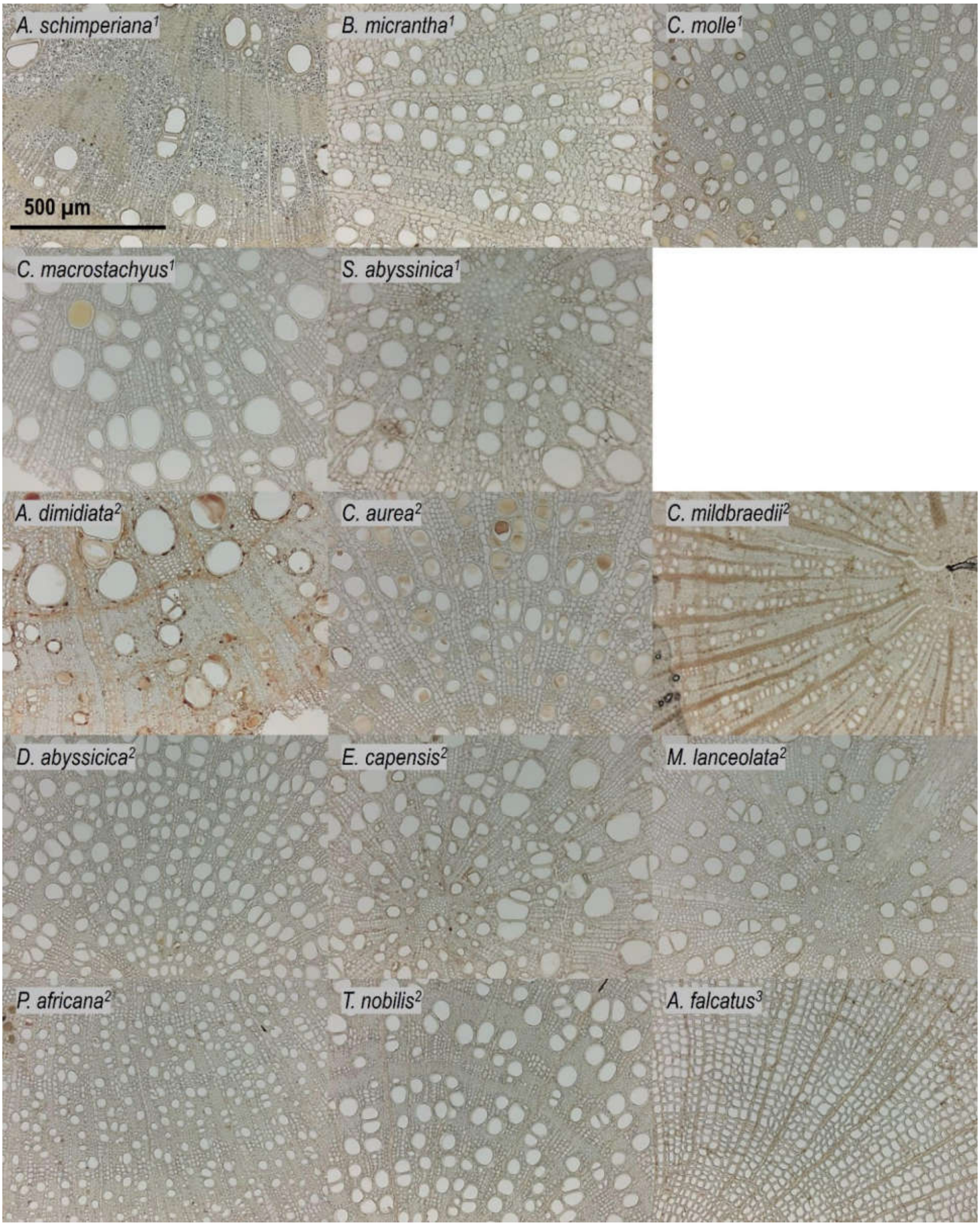

Supplementary Figure S1. Xylem anatomy of coarse roots of 14 woody species in a seasonally dry Ethiopian Highland forest. Species are 13 deciduous[1] or evergreen[2] woody angiosperms, and the gymnosperm[3] *A. falcatus*; see Table 1 for details.

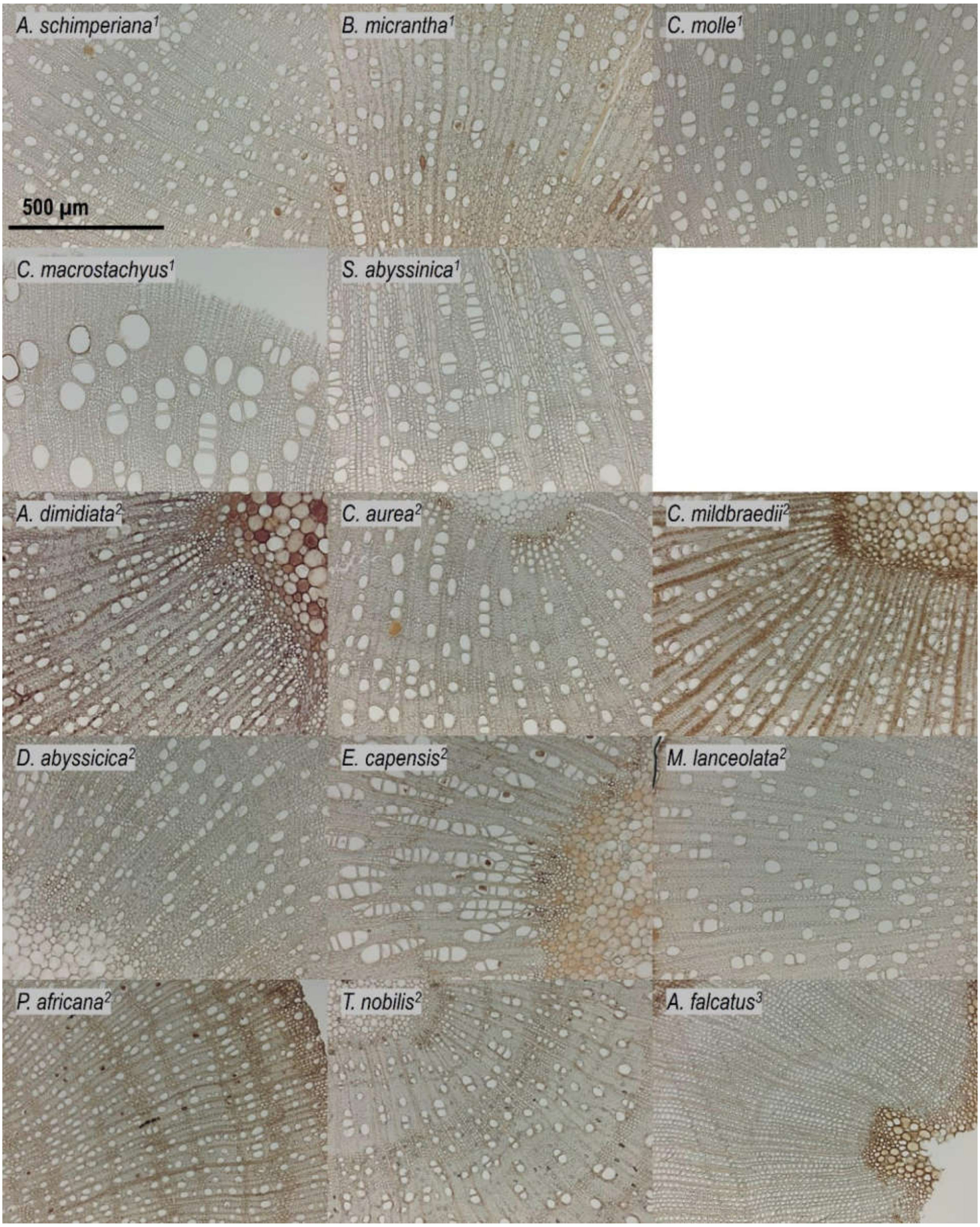

Supplementary Figure S2. Xylem anatomy of 2nd-year branches of 14 woody species in a seasonally dry Ethiopian Highland forest. Species are 13 deciduous[1] or evergreen[2] woody angiosperms, and the gymnosperm[3] *A. falcatus*; see Table 1 for details.

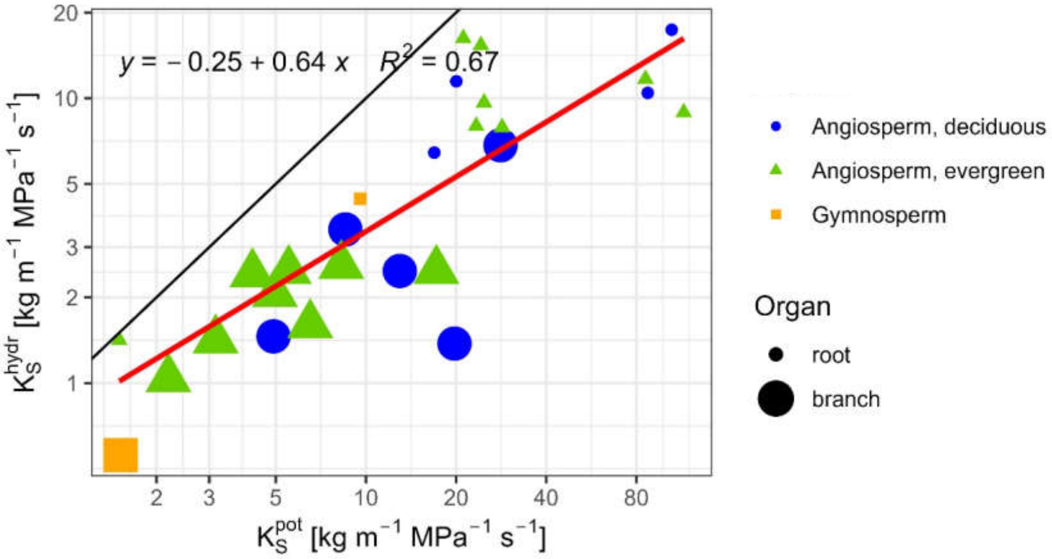

Supplementary Figure S3. Linear regression (red line) between potential (K_S^{pot}) and measured (K_S^{hydr}) conductivities in roots (small symbols), and branches (large symbols) of 14 species in a seasonally dry Ethiopian Highland forest (means per species); see Table 1 for details. A 1:1 line is given in black. Note: logarithmic scales were used to improve the display; the gymnosperm was excluded from the regression.
